# Fiber-type vulnerability and proteostasis reprogramming in skeletal muscle during pancreatic cancer cachexia

**DOI:** 10.1172/jci.insight.200396

**Published:** 2026-01-27

**Authors:** Bowen Xu, Aniket S. Joshi, Meiricris Tomaz da Silva, Silin Liu, Ashok Kumar

**Affiliations:** 1 Institute of Muscle Biology and Cachexia, University of Houston College of Pharmacy, Houston, Texas, USA.; 2 Department of Biomedical engineering, Cullen College of Engineering, University of Houston, Houston, Texas, USA.; 3 Department of Pharmacological and Pharmaceutical Sciences, University of Houston College of Pharmacy, Houston, Texas, USA.

**Keywords:** Cell biology, Muscle biology, Cancer, Expression profiling, Muscle

## Abstract

Cachexia is a debilitating syndrome characterized by progressive skeletal muscle wasting, commonly affecting patients with cancer, particularly those with pancreatic cancer. Despite its clinical significance, the molecular mechanisms underlying cancer cachexia remain poorly understood. In this study, we utilized single-nucleus RNA-seq (snRNA-seq) and bulk RNA-seq, complemented by biochemical and histological analyses, to investigate molecular alterations in the skeletal muscle of the KPC mouse model of pancreatic cancer cachexia. Our findings demonstrated that KPC tumor growth induced myofiber-specific changes in the expression of genes involved in proteolytic pathways, mitochondrial biogenesis, and angiogenesis. Notably, tumor progression enhanced the activity of specific transcription factors that regulate the mTORC1 signaling pathway, along with genes involved in translational initiation and ribosome biogenesis. Skeletal muscle–specific, inducible inhibition of mTORC1 activity further exacerbated muscle loss in tumor-bearing mice, highlighting its protective role in maintaining muscle mass. Additionally, we uncovered new intercellular signaling networks within the skeletal muscle microenvironment during pancreatic cancer–induced cachexia. Our study reveals previously unrecognized molecular mechanisms that regulate skeletal muscle homeostasis, and it identifies potential therapeutic targets for the treatment of pancreatic cancer–associated cachexia.

## Abstract 2

This study identified previously unrecognized molecular mechanisms and intercellular communications that drive skeletal muscle wasting during pancreatic cancer-associated cachexia.

## Graphical abstract

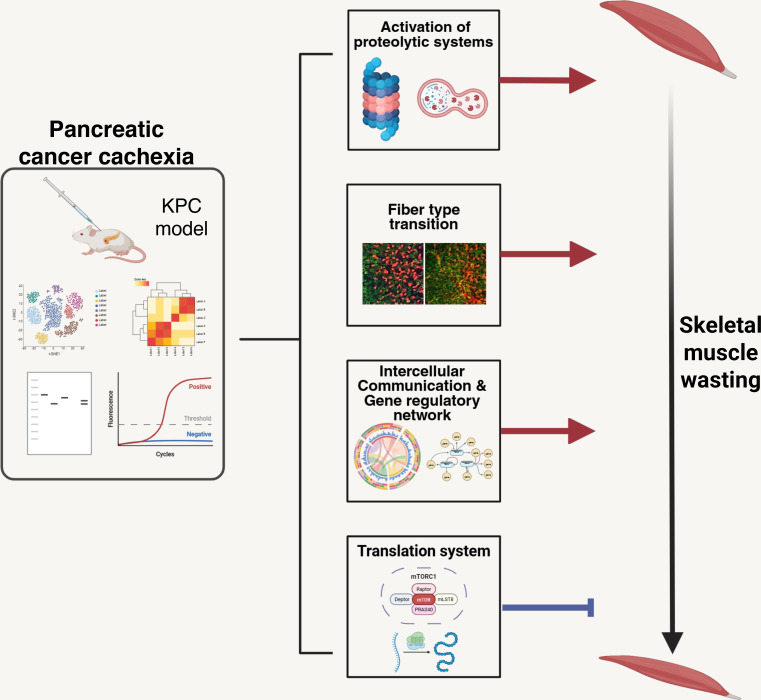

## Introduction

Cancer cachexia is a multifactorial syndrome characterized by a significant loss of skeletal muscle mass, often accompanied by adipose tissue wasting. It contributes to reduced responsiveness to antineoplastic therapies, diminished quality of life, and lower overall survival in patients with cancer (1). While cachexia is common in individuals with gastroesophageal, lung, and head and neck cancers, it is particularly prevalent in pancreatic ductal adenocarcinoma (PDAC), where it is present in approximately 60% of patients at diagnosis and up to 85% in advanced stages (1–5). Despite its high prevalence and clinical significance, there are currently no effective therapies approved for the prevention or treatment of cancer cachexia, partly because etiology of cancer-induced cachexia remains less understood (4).

Disruption of proteostasis is one of the important mechanisms leading to skeletal muscle wasting in many catabolic conditions, including cancer (3, 6, 7). Many proinflammatory cytokines and tumor-derived factors increase muscle proteolysis through stimulating ubiquitin-proteasome system (UPS) and autophagy (8). In addition to increased proteolysis, other pathological changes also occur in skeletal muscle during cancer cachexia. For example, mitochondrial dysfunction resulting in reduced oxidative phosphorylation (OXPHOS) and heightened oxidative stress are common pathological features of skeletal muscle in animal models and patients with cachectic cancer (1, 9). Cancer cachexia also involves disruption of neuromuscular junctions (NMJs), leading to functional denervation and the loss of skeletal muscle mass and function (10, 11). Furthermore, endothelium dysfunction and loss of skeletal muscle vascular density are common features of multiple animal models and in patients with cancer cachexia (12).

Although it is generally believed that the rate of protein synthesis is reduced in skeletal muscle during cancer progression, some studies have shown that protein synthesis remains unaffected or even increases in animal models and patients with cancer cachexia (13). In mammalian cells, translation is initiated by the binding of eukaryotic initiation factor 4E (eIF4E) to the 5′ cap of the mRNA, followed by the recruitment of eIF4G, eIF4A, and eIF4B. This assembly forms the eIF4F complex, also known as the translation preinitiation complex (14). The mechanistic target of rapamycin complex 1 (mTORC1)- p70 ribosomal S6 kinase (p70S6K) is a major signaling mechanism that stimulates translation initiation in mammalian cells, including skeletal muscle in response to many growth factors and physical exercise (7). While mTORC1-mediated signaling is not required for the maintenance of muscle mass, it plays an important role in regulating mitochondrial proteome and muscle contractile force production in response to Akt-mediated myofiber hypertrophy (15). Intriguingly, recent studies employing genetic mouse models have demonstrated that constitutive activation of mTORC1 results in muscle wasting and myopathy without having any significant effect on protein synthesis (7, 16, 17). However, the role of mTORC1-mediated signaling in the regulation of muscle mass during pancreatic cancer cachexia remains unknown.

While myofibers are the major cell type that are present in skeletal muscle, there are many other cell types, including fibroblasts, endothelial cells, and inflammatory immune cells, that may also be affected and contribute to the loss of muscle mass during cancer cachexia (18). Because of their relative low abundance, until recently, it was difficult to understand the molecular changes that occur in individual cell types within skeletal muscle during various pathological conditions, including cancer. Recent studies using single nucleus RNA-seq (snRNA-seq) have deciphered the role of a few molecular pathways that may regulate muscle mass during cancer cachexia (11, 18, 19). However, the molecular and signaling mechanisms that regulate proteostasis and intercellular communications within skeletal muscle microenvironment during pancreatic cancer cachexia remain poorly understood.

A recent transcriptomic study has identified several molecular and signaling alterations in the skeletal muscle of male patients with pancreatic cancer, especially at the late stage of the disease, that closely resemble those observed in KRAS^G120^P53^R172H^Pdx-Cre^+/+^ (KPC) tumor–bearing mice, a well-established mouse model of pancreatic cancer cachexia (20–22). In the present study, we employed snRNA-seq and bulk RNA-seq, along with various biochemical and histological techniques, to investigate cell type-specific alterations in the skeletal muscle of KPC tumor–bearing mice. Our results demonstrate that KPC tumor growth causes fiber-type reprograming and distinctly affects the activation of specific catabolic and anabolic signaling and proteostasis in skeletal muscle of adult mice. We also demonstrate that mTORC1 signaling, translational initiation, and ribosome biogenesis are significantly upregulated in fast-type myofibers of KPC tumor–bearing mice. Targeted inhibition of mTORC1 signaling in adult mice exacerbates muscle wasting in response to tumor growth. Finally, our snRNA-seq transcriptome analysis has identified previously unknown intercellular communications that may play important roles in muscle wasting during pancreatic cancer cachexia.

## Results

### Single-nucleus RNA-seq reveals cellular heterogeneity in skeletal muscle during pancreatic cancer cachexia.

**Figure 1:**
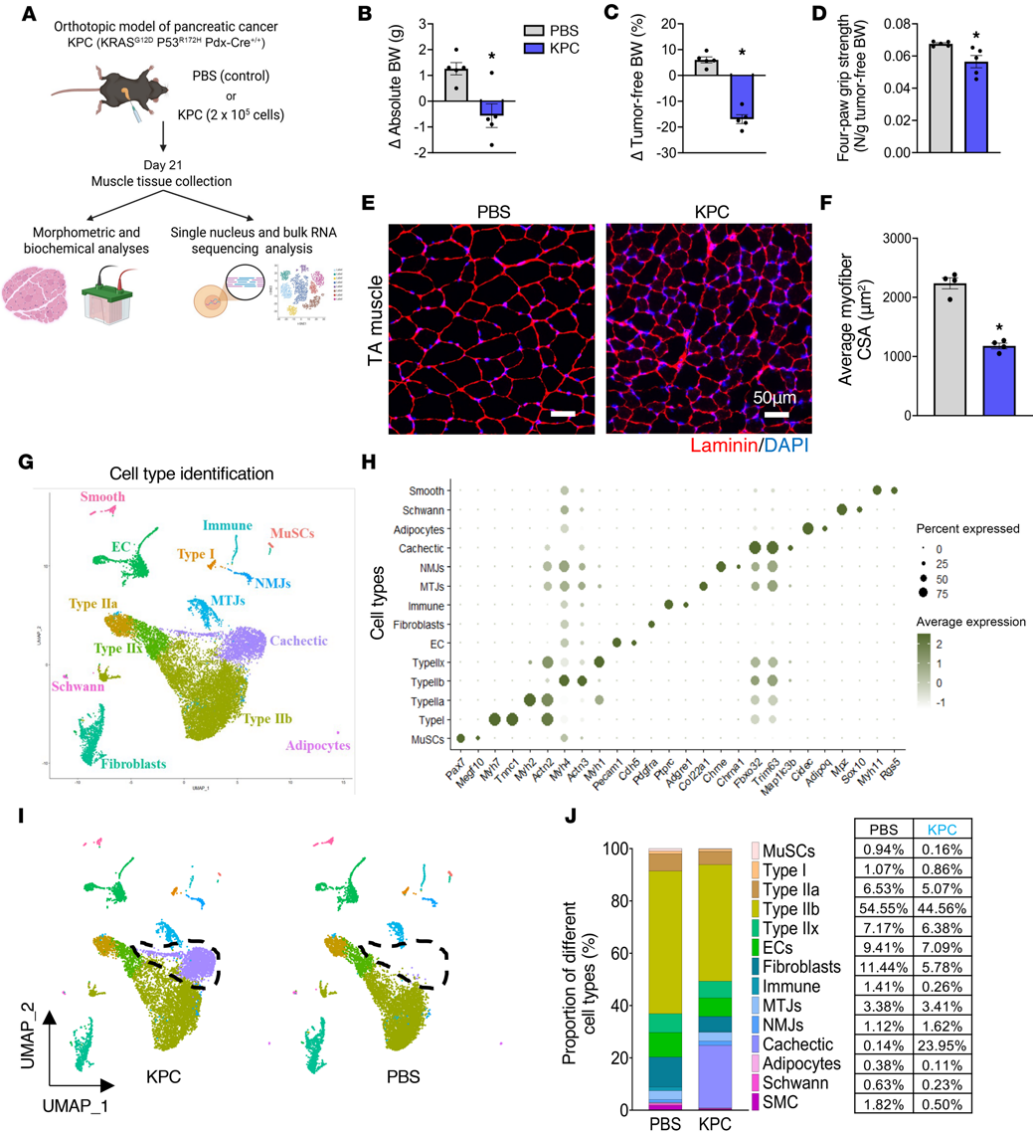
Cellular diversity in skeletal muscle from control and KPC tumor–bearing mice. (**A**) Schematic representation of experimental design. Twelve-week-old male C57BL/6 were injected with KPC cells or PBS into the pancreas. On day 21, hindlimb muscles of the mice were isolated and processed for morphometric, biochemical, and snRNA-Seq analyses. (**B** and **C**) Changes in absolute body (BW) weight (**B**), and tumor-free BW (**C**) in control and KPC tumor–bearing mice. (**D**) Four-paw grip strength normalized by tumor-free BW of control and KPC tumor–bearing mice. (**E**) Representative anti-laminin– and DAPI-stained TA muscle transverse sections. Scale bar: 50 μm. (**F**) Quantification of average myofiber cross-sectional area (CSA) in TA muscle of control and KPC tumor–bearing mice. *n* = 5 per group. All data are presented as mean ± SEM. *P ≤ 0.05, significantly different from control mice injected with PBS alone analyzed by unpaired Student’s *t* test. (**G**) UMAP plot representing manually annotated clusters for cell type identity. (**H**) Expression of representative marker genes define major cell types in dot plot. (**I**) Split-UMAP illustrating transcriptomic clustering of muscle-derived nuclei of control and KPC tumor–bearing mice. (**J**) Proportion of different nuclei population in GA muscle of control and KPC tumor–bearing mice.

To define transcriptional alterations in skeletal muscle during pancreatic cancer–induced cachexia, we used an orthotopic KPC mouse model (20). Briefly, 2 × 10^5^ KPC cells were injected into the pancreatic tail of 12-week-old WT mice, while control mice received PBS. Twenty-one days after injection, mice were assessed for body weight and grip strength, followed by collection of hindlimb muscles for histological, biochemical, and transcriptomic analyses (Figure 1A). Compared with controls, KPC tumor–bearing mice exhibited significant reductions in absolute and tumor-free body weight (Figure 1, B and C), as well as decreased 4-paw grip strength normalized by tumor-free body weight (Figure 1D). Histological analysis of tibialis anterior (TA) muscle revealed a significant reduction in average myofiber cross-sectional area (CSA), consistent with muscle atrophy (Figure 1, E and F).

We next performed single-nucleus RNA-seq (snRNA-seq) on pooled gastrocnemius (GA) muscle from 5 control and 5 KPC tumor–bearing mice. After quality control, 8,953 nuclei from control, and 9,794 nuclei from KPC samples were retained for analysis. Unsupervised clustering of the integrated dataset identified 21 clusters, which were annotated into 13 distinct cell types based on established marker genes (11, 19, 23), including myonuclear subtypes, muscle stem cells, endothelial cells, fibroblasts, immune cells, and other resident populations (Figure 1, G and H, and Supplemental Figure 1A; supplemental material available online with this article; https://doi.org/10.1172/jci.insight.200396DS1). Notably, 2 nuclear clusters (clusters 2 and 14), hereafter termed Cachectic-1 and Cachectic-2, were detected exclusively in KPC tumor–bearing mice (Figure 1I). These clusters were characterized by strong enrichment of the muscle-specific E3 ubiquitin ligases Fbxo32 (MAFbx) and Trim63 (MuRF1) and were therefore annotated as “cachectic” myonuclei (Figure 1, G and H, and Supplemental Figure 1A). Similar multicluster patterns were observed for Type IIb myonuclei, fibroblasts, and endothelial cells, indicating heterogeneity within these populations. Comparative pathway analysis revealed that Cachectic-1 and Cachectic-2 clusters shared activation of KEAP1/NFE2L2 signaling and catabolic and proteolytic pathways, while also exhibiting distinct features: Cachectic-1 was enriched for ER protein processing and TOR signaling, whereas Cachectic-2 was associated with nutrient sensing and glycogen metabolism (Supplemental Figure 1B). Further analysis of Type IIb myonuclei uncovered functional diversity across 4 subclusters, with enrichment for OXPHOS, cytoskeletal organization, proteolysis/ER stress, or hormone-mediated signaling (Supplemental Figure 1C). Although Type IIb fibers are classically glycolytic, a subset of Type IIb myonuclei displayed elevated OXPHOS gene expression, consistent with emerging evidence for metabolically distinct Type IIb subtypes (24). Finally, analysis of cell type proportions revealed that cachectic myonuclei constituted ~24% of total nuclei in KPC samples and were absent in controls, resulting in an apparent reduction in the relative abundance of most other cell types, except myotendinous and NMJ-associated nuclei (Figure 1J). Collectively, these data demonstrate pronounced cellular and transcriptional heterogeneity in skeletal muscle during pancreatic cancer cachexia, including the emergence of distinct cachectic myonuclear populations.

### Pancreatic cancer induces a robust atrophy program in skeletal muscle.

**Figure 2:**
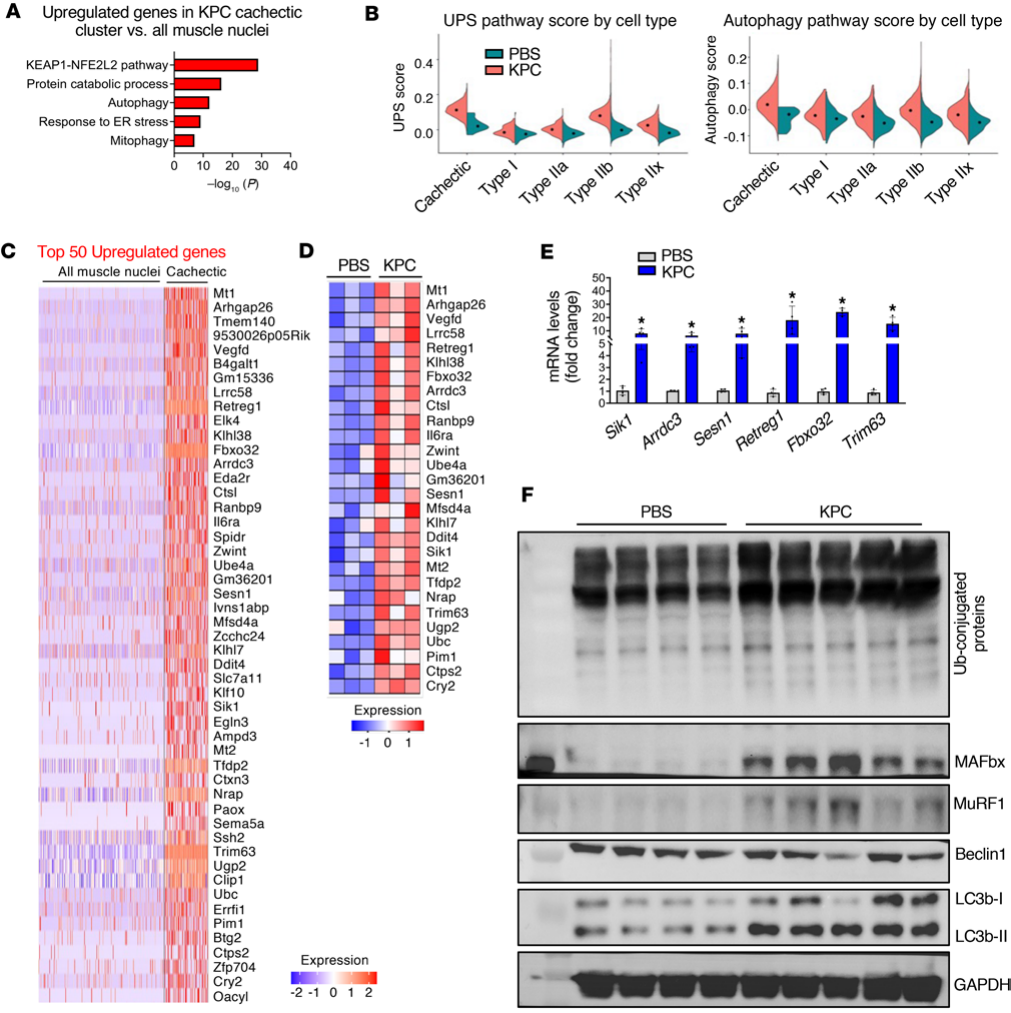
Upregulation of proteolytic systems in cachectic myofibers. (**A**) Enrichment analysis of upregulated genes in cachectic myonuclei highlighting significantly enriched pathways related to protein degradation. (**B**) Violin plots showing module scores for the ubiquitin-proteasome system (UPS) and autophagy across different myonuclear clusters from GA muscle of control and KPC tumor–bearing mice. Scores were calculated using Seurat’s scoring function to quantify pathway activity. Each violin represents the distribution of pathway scores within a fiber type. (**C**) Heatmap displaying the expression of the top 50 upregulated genes in cachectic nuclei compared with all the myonuclei in control mice. (**D**) Heatmap showing the expression of representative top 50 upregulated genes from Bulk RNA-seq at tissue level. (**E**) Independent qPCR analysis of the upregulated top-ranked representative genes in GA muscle of control and KPC tumor–bearing mice. (**F**) Immunoblots of the levels of total ubiquitinated proteins, MAFbx, MuRF1, Beclin1, LC3B-I/II, and unrelated protein GAPDH in TA muscle of control and KPC tumor bearing mice. *n* = 4 for PBS group and *n* = 5 in KPC tumor group. All data are presented as mean ± SEM. *P ≤ 0.05, values significantly different from PBS-injected control mice analyzed by unpaired Student’s *t* test.

To define the transcriptional features of the cachectic cluster in KPC tumor–bearing mice, we performed differential gene expression analysis comparing cachectic myonuclei with all myonuclei from control mice (Type I, Type IIa, Type IIb, Type IIx, MTJ, NMJ, and MuSC), thereby enriching for muscle-specific responses to tumor growth. Using a threshold of |log_2_FC| > 0.58 and *P* < 0.05, we identified 4,360 differentially expressed genes (DEGs), including 1,811 upregulated and 2,549 downregulated genes (Supplemental Figure 2A). Pathway enrichment analysis revealed strong upregulation of genes associated with the KEAP1/NFE2L2 (NRF2) pathway, protein catabolism, autophagy, mitophagy, and endoplasmic reticulum (ER) stress responses in cachectic myonuclei relative to controls (Figure 2A). The KEAP1/NRF2 pathway is a well-established protective mechanism activated by oxidative stress in skeletal muscle during cancer cachexia, and muscle-specific loss of NRF2 has been shown to exacerbate wasting (25). Consistent with enhanced proteolysis, gene set scoring analysis demonstrated elevation of UPS and autophagy pathway scores in cachectic and Type IIb myonuclear clusters, with more moderate increases in Type IIa and IIx clusters and modest changes in Type I clusters, indicating fiber-type–specific activation of atrophy pathways (Figure 2B and Supplemental Figure 2, B and C). These findings were supported by bulk RNA-seq analysis, which showed significant upregulation of multiple UPS- and autophagy/mitophagy-related genes in skeletal muscle from KPC tumor–bearing mice (26). Notably, several established atrophy-associated genes, including *Fbxo32*, *Trim63*, *Eda2r*, *Il6ra*, and *Ddit4*, were among the top 50 upregulated genes in cachectic myonuclei and were similarly enriched at the tissue level, as illustrated in the bulk RNA-seq heatmap (Figure 2, C and D). Validation by qPCR confirmed significant increases in *Sik1*, *Arrdc3*, *Sesn1*, *Retreg1*, *Fbxo32*, and *Trim63* mRNA levels in skeletal muscle of KPC tumor–bearing mice compared with control mice (Figure 2E). At the protein level, Western blot analysis revealed increased abundance of ubiquitin-conjugated proteins, MAFbx, MuRF1, and LC3B-II, but not Beclin1, in TA muscle from KPC tumor–bearing mice (Figure 2F and Supplemental Figure 2D). Together, these transcriptomic and biochemical analyses demonstrate that KPC tumor growth activates a coordinated skeletal muscle atrophy program characterized by robust induction of UPS- and autophagy-mediated proteolysis in cachectic myonuclei.

### KPC tumor growth suppresses mitochondrial metabolism, angiogenesis, and structural maintenance programs in skeletal muscle.

**Figure 3:**
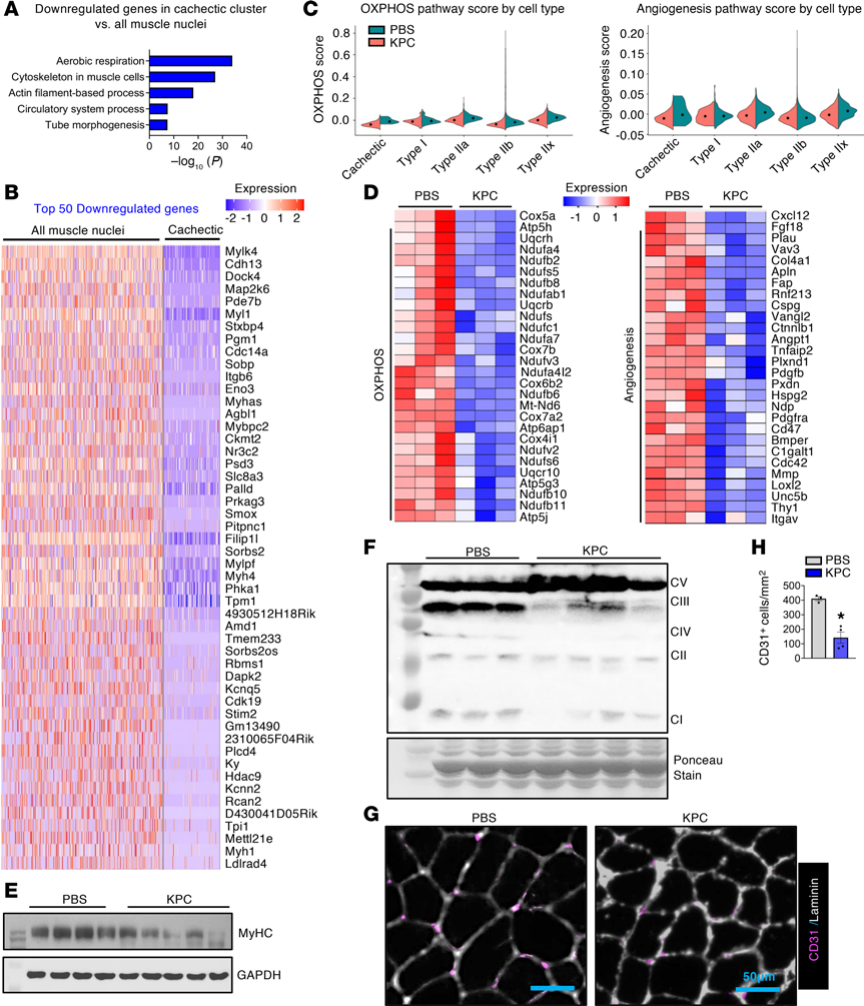
Downregulation of oxidative phosphorylation and angiogenesis pathway in cachectic myofibers. (**A**) Enrichment analysis of downregulated genes in cachectic myonuclei highlighting significantly enriched pathways. (**B**) Heatmap displaying the expression of the top 50 downregulated genes in cachectic nuclei compared with all the myonuclei of control mice. (**C**) Violin plots showing module scores for the OXPHOS and angiogenesis processes across different myonuclear clusters from GA muscle of control and KPC tumor–bearing mice. Each violin represents the distribution of pathway scores within a fiber type. (**D**) Heatmap showing the expression of representative genes related to OXPHOS and Angiogenesis from bulk RNA-seq analysis of GA muscle of control and KPC tumor–bearing mice. (**E** and **F**) Immunoblots showing protein levels of MyHC (**E**), and OXPHOS (**F**) complexes I–V in skeletal muscle of control and KPC tumor–bearing mice. (**G** and **H**) Representative photomicrographs (**G**) and quantification of CD31^+^ cells (**H**) per unit area after immunostaining for CD31 (pink) and Laminin (white) protein in soleus muscle from control and KPC tumor–bearing mice. Scale bar: 50 μm. *n* = 3–4 per group. All data are presented as mean ± SEM. *P ≤ 0.05, values significantly different from control mice analyzed by unpaired Student’s *t* test.

We next analyzed the downregulated genes in cachectic nuclear cluster of KPC tumor–bearing mice. Pathway enrichment analysis showed that genes involved in aerobic respiration, muscle cytoskeletal organization, actin filament–based processes, circulatory system development, and tube morphogenesis were significantly downregulated in cachectic myonuclei compared with all myonuclei from control mice (Figure 3A). Consistently, analysis of the top 50 downregulated genes revealed repression of multiple genes essential for muscle structure and contraction, including *Myl1*, *Myh4*, *Myh1*, *Tpm1*, *Mylk4*, *Mylpf*, and *Itgb6*, in cachectic myonuclei (Figure 3B). Gene set scoring further demonstrated a significant reduction in OXPHOS-related gene expression across cachectic and other myonuclear clusters, independent of muscle fiber type, in KPC tumor–bearing mice. Angiogenesis scores were also reduced in cachectic and most other myonuclear clusters, with the exception of the Type I myonuclear cluster (Figure 3C and Supplemental Figure 3, A and B). Analysis of bulk RNA-seq data from GA muscle corroborated these findings, showing coordinated downregulation of OXPHOS- and angiogenesis-related genes in KPC tumor–bearing mice compared with controls (Figure 3D). These transcriptional changes were validated by performing biochemical or histological analysis. Western blotting revealed a significant reduction in myosin heavy chain (MyHC) as well as mitochondrial respiratory chain complexes I, III, and IV in skeletal muscle from KPC tumor–bearing mice (Figure 3, E and F, and Supplemental Figure 3, C and D). In parallel, immunostaining of soleus muscle sections demonstrated a significant decrease in CD31^+^ vascular density, indicating impaired angiogenesis (Figure 3, G and H). Collectively, these results indicate that pancreatic cancer–associated cachexia is characterized by broad repression of mitochondrial oxidative capacity, vascularization, and myofiber structural and contractile programs in skeletal muscle.

### KPC tumor growth induces fiber-type reprograming in skeletal muscle.

**Figure 4:**
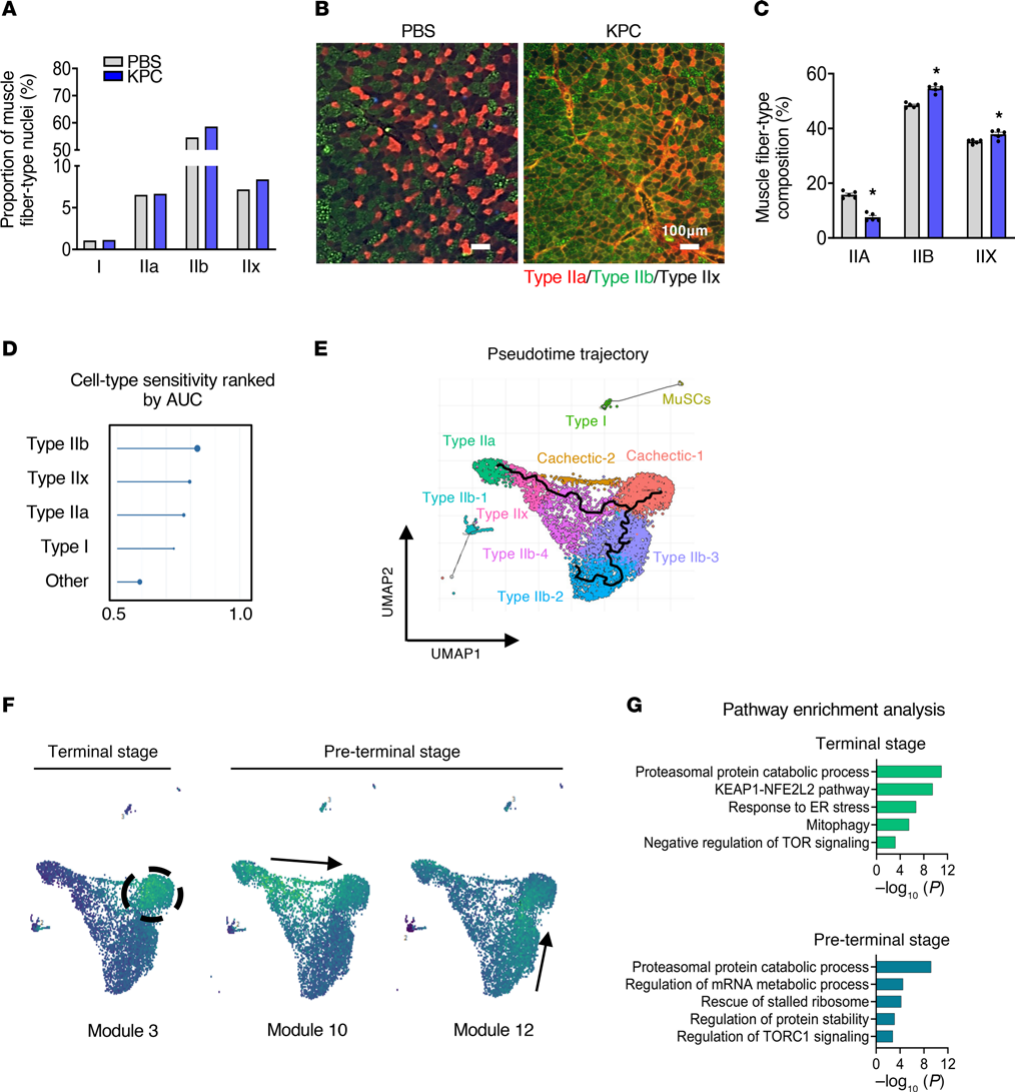
KPC tumor growth causes fiber-type remodeling in skeletal muscle. (**A**) Proportion of muscle fiber-type nuclei in snRNA-seq dataset. (**B**) Representative cross-sections of TA muscle from control and KPC tumor–bearing mice after immunostaining for myosin heavy chain (MyHC) isoforms to distinguish fiber types: Type IIa fibers (red), Type IIb fibers (green), and Type IIx fibers (black). Scale bar: 100 μm. (**C**) Quantification of fiber-type composition in TA muscle of control and KPC tumor–bearing mice. *n* = 4 per group. Data are presented as mean ± SEM. *P ≤ 0.05, values significantly different from control mice analyzed by unpaired Student’s *t* test. (**D**) Augur-based analysis of perturbation sensitivity of different fiber-type in skeletal muscle of control and KPC tumor–bearing mice. (**E**) Pseudotime trajectory and annotation of myonuclei subpopulations by UMAP. (**F**) UMAP visualization of module-specific gene expression across myonuclear clusters. (**G**) Enrichment analysis of the pathways associated with the preterminal/terminal stage gene modules.

Skeletal muscle fibers are heterogeneous in their expression of MyHC isoforms, which define distinct contractile and metabolic properties and allow classification into Type I, IIa, IIx, and IIb fibers (27). Although prior studies have reported variable effects of cancer cachexia on fiber-type composition, including shifts toward more glycolytic phenotypes (28–30), the effect of pancreatic cancer–induced cachexia remains incompletely defined. To determine whether KPC tumor growth alters fiber-type composition and associated transcriptional programs, we first analyzed the distribution of Type I, IIa, IIx, and IIb myonuclei in our snRNA-seq dataset. To avoid bias arising from the emergence of cachectic-specific clusters, these nuclei were excluded prior to analysis. This analysis revealed a relative increase in Type IIx and Type IIb myonuclei in KPC tumor–bearing mice compared with controls (Figure 4A). Consistent with these findings, IHC analysis of TA muscle demonstrated a significant reduction in Type IIa fibers accompanied by a corresponding increase in Type IIx and IIb fibers in KPC tumor–bearing mice (Figure 4, B and C). We next assessed fiber-type–specific vulnerability to cachexia using the machine learning-based Augur approach. This analysis ranked Type IIb myonuclei as the most transcriptionally perturbed in response to tumor growth, followed by Type IIx and IIa, whereas Type I myonuclei were least affected (Figure 4D). Together, these results indicate that KPC tumor growth drives a fiber-type transition from Type IIa toward more glycolytic Type IIx/IIb phenotypes and that Type IIb myofibers exhibit the greatest susceptibility to KPC tumor–induced atrophy.

To further define the transcriptional dynamics underlying the transition of myonuclei toward a cachectic state, we performed trajectory analysis to model myonuclear state transitions along a pseudotime axis representing cachexia progression. The inferred trajectory originated from both Type IIa and Type IIb myonuclear clusters and converged on the terminal cachectic cluster. In contrast, Type I myonuclei showed no trajectory connection to the cachectic state, reinforcing the notion that Type I myofibers are relatively resistant to wasting during pancreatic cancer cachexia (Figure 4E).

Because skeletal muscle from KPC tumor–bearing mice likely contains myofibers at multiple stages of atrophy, we next sought to identify shared and stage-specific transcriptional programs by defining coregulated gene expression modules across myonuclear clusters. Using Louvain community detection, we identified 41 coregulated gene modules (Supplemental Figure 4). Several modules displayed cachexia-associated activity patterns consistent with either “preterminal” (transitional) or “terminal” (fully cachectic) states (Figure 4F). Notably, Module 3 was selectively enriched in cachectic myonuclei and represented a terminal-stage program characterized by activation of canonical atrophy pathways, including proteasome-mediated protein degradation, KEAP1/NFE2L2 signaling, ER stress responses, mitophagy, and negative regulation of mTOR signaling (Figure 4, F and G). In contrast, Modules 10 and 12 peaked at a preterminal divergence point along the trajectory and were enriched for genes involved in proteasomal protein catabolism, mRNA metabolism, ribosome rescue, protein stability, and regulation of TORC1 signaling, suggesting distinct transcriptional programs that preceded overt cachexia (Figure 4, F and G). Together, these analyses delineate a progressive, fiber-type–dependent transcriptional trajectory from healthy to cachectic myonuclei during pancreatic cancer cachexia.

**Figure 5:**
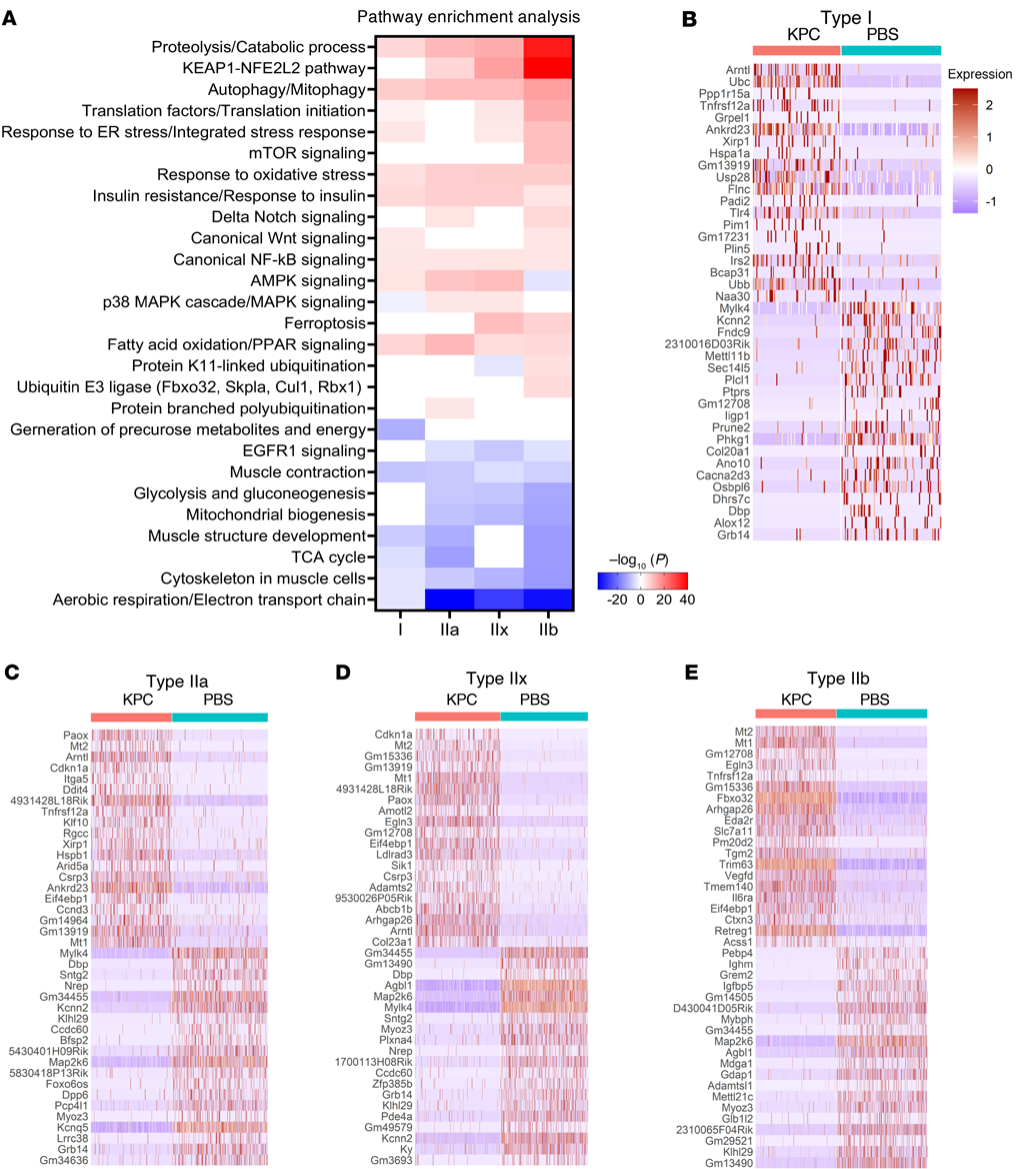
Transcriptome analysis of different myofiber types in control and KPC tumor–bearing mice. (**A**) Heatmap showing the pathway enrichment analysis for the DEGs across individual myofiber nuclear cluster. (**B**–**E**) The heatmap illustrates the expression of the top 20 upregulated and top 20 downregulated genes in the nuclear cluster of Type I (**B**), Type IIa (**C**), Type IIx (**D**), and Type IIb (**E**) myofibers.

We next examined fiber-type–specific transcriptional responses to KPC tumor growth by analyzing differential gene expression within individual myonuclear populations from control and tumor-bearing mice. Pathway enrichment analysis revealed several commonly upregulated pathways across all 4 myonuclear types, including proteolysis, regulation of catabolic processes, autophagy/mitophagy, fatty acid oxidation and PPAR signaling, oxidative stress responses, insulin resistance, and canonical NF-κB signaling (Figure 5A). In addition to these shared responses, distinct fiber-type–specific regulatory programs were evident. AMPK signaling was upregulated in Type I, IIa, and IIx myonuclei but downregulated in Type IIb myonuclei, whereas KEAP1/NFE2L2 signaling was selectively induced in Type IIa, IIx, and IIb clusters but not in Type I myonuclei. Notably, genes encoding translation factors and components of translational initiation were significantly elevated in Type IIb myonuclei relative to other fiber types. Consistent with this heightened remodeling, mTOR signaling, K11-linked ubiquitination, and ubiquitin E3 ligase pathways were significantly enriched exclusively in Type IIb myonuclei (Figure 5A). Analysis of downregulated pathways revealed that aerobic respiration and muscle contraction programs were broadly suppressed across all fiber types. In contrast, pathways related to mitochondrial biogenesis, glycolysis/gluconeogenesis, and EGFR1 signaling were selectively downregulated in all Type II myonuclear clusters but remained largely unchanged in Type I myonuclei (Figure 5A).

The top 20 upregulated and downregulated genes within each fiber type highlighted both shared and fiber-type–specific gene regulation (Figure 5, B–E). Among these, *Tnfrsf12a* (Fn14), the receptor for the catabolic cytokine TWEAK, was prominently upregulated in Type IIa and Type IIb myonuclei, consistent with its established role in cancer cachexia–induced muscle wasting (31, 32). Similarly, *Eda2r*, another mediator of muscle atrophy (33), was highly enriched in Type IIb myonuclei. Importantly, the canonical atrogenes *Fbxo32* (MAFbx) and *Trim63* (MuRF1) were among the top 20 upregulated genes specifically in Type IIb myonuclei, reinforcing the heightened vulnerability of this fiber type to pancreatic cancer–associated wasting. Additional genes, including *Mt1* and *Mt2*, were strongly induced in Type IIa, IIx, and IIb clusters; however, their functional roles in cachexia remain unknown (Figure 5, B–E). Together, these findings demonstrate that KPC tumor growth drives fiber-type–specific transcriptional reprogramming, with Type IIb myofibers exhibiting the most pronounced activation of catabolic and remodeling pathways during pancreatic cancer cachexia.

### Transcriptional programs governing protein synthesis are altered in myofibers during pancreatic cancer cachexia.

**Figure 6:**
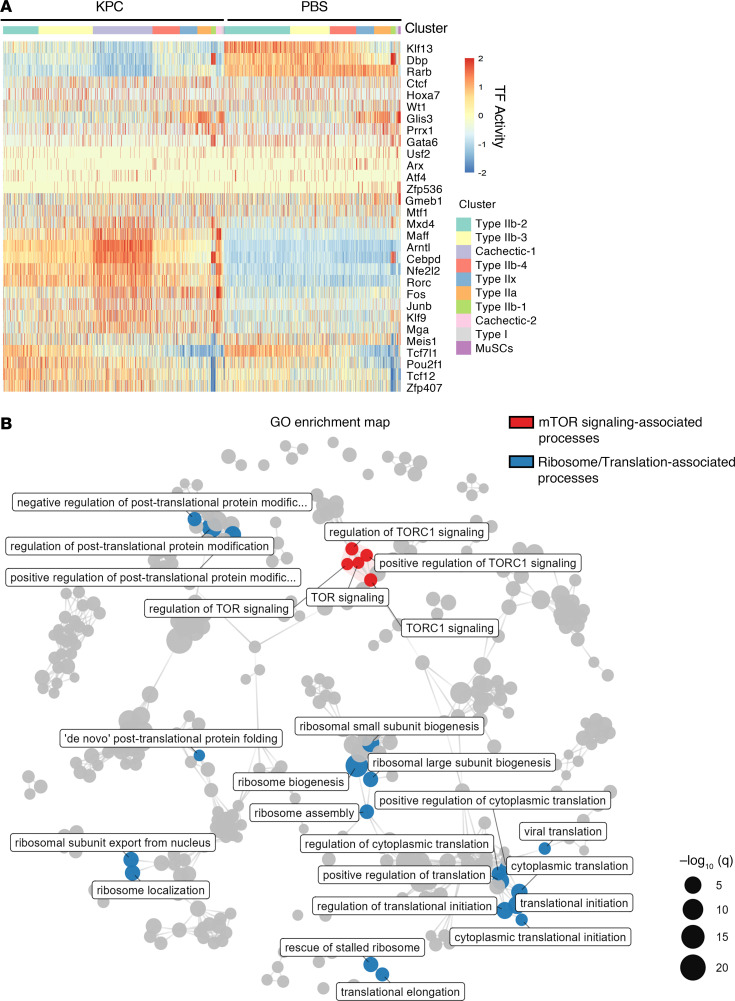
Transcriptional rewiring in cachectic muscle of KPC tumor–bearing mice. (**A**) Heatmap of regulon activity inferred by SCENIC across myonuclei. (**B**) Gene ontology (GO) enrichment network of high-confidence TF targets, with core mTOR signaling terms annotated in red and translation/ribosome-related terms in blue.

Because transcription factors (TFs) act as master regulators of gene expression, we next examined how TF activity is altered in skeletal muscle during pancreatic cancer cachexia. To define transcriptional regulatory programs associated with cachexia, we computationally inferred TF activity and gene regulatory networks from our snRNA-seq dataset. DEGs in myonuclear clusters were first analyzed for overrepresented TF-binding motifs. These data were then integrated with gene expression using the Single-Cell rEgulatory Network Inference and Clustering (SCENIC) framework to infer TF regulons and quantify regulon activity at single-nucleus resolution using AUCell. This analysis identified multiple TFs whose activity was significantly altered in response to KPC tumor growth. A heatmap of the 15 most strongly affected TFs revealed both increased and decreased regulon activity across myonuclear clusters, with pronounced changes in cachectic myonuclei (Figure 6A). To determine the functional relevance of these TFs, we performed pathway enrichment and network-based clustering on their target genes. This revealed several interconnected functional modules, notably including processes related to regulation of target of rapamycin complex 1 (TORC1) signaling (highlighted in red) and a broader network of translation- and ribosome-associated pathways (highlighted in blue) (Figure 6B).

To prioritize TFs most relevant to cachexia-associated remodeling, we applied 3 selection criteria: (a) increased regulon activity inferred by SCENIC/AUCell, (b) elevated TF expression in cachectic myonuclei, and (c) significant overlap between TF regulons and genes upregulated in cachectic clusters. Candidate TFs were further filtered based on their association with TORC1- and translation-related processes. This approach identified TFs with distinct activity patterns, including factors whose activity was largely restricted to cachectic myonuclei (e.g., *Foxo1*, *Clock*, *Elk4*) and others showing broader induction across myonuclear populations (e.g., *Zbtb20*, *Tead1*), suggesting a hierarchical organization of transcriptional control (Supplemental Figure 5).

**Figure 7:**
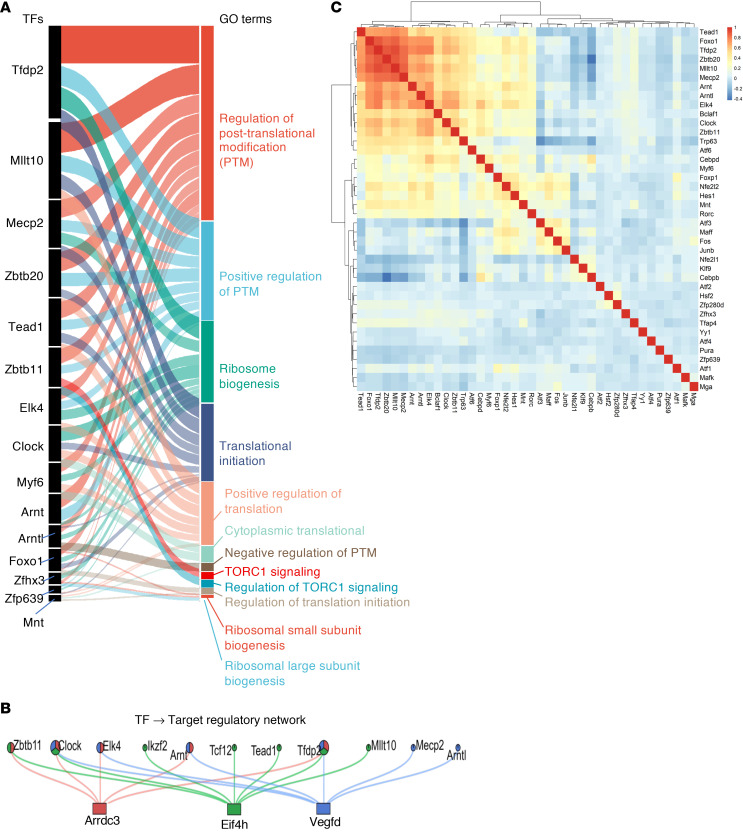
Identification of mTORC1 as a target of transcriptional network in cachectic muscle. (**A**) Alluvial diagram linking TFs to enriched GO terms. Ribbon width reflects the number of shared target genes. (**B**) Convergence of enriched TFs regulatory network at key nodes/target genes. (**C**) Correlation matrix of regulon activity scores among deregulated TFs in cachectic myofibers of KPC tumor–bearing mice.

An alluvial diagram illustrates the relationships between prioritized TFs (Tfdp2, Mllt10, Mecp2, Zbtb20, Tead1, Zbtb11, Elk4, Clock, Myf6, Arnt, Arntl, Foxo1, Zfhx3, Zfp639, and Mnt) and their associated Gene Ontology terms, with ribbon width reflecting the extent of target gene overlap (Figure 7A). Notably, multiple TFs converged on key biological processes, including ribosome biogenesis, translational initiation, and regulation of posttranslational protein modification. Because TORC1 is a central regulator of protein synthesis and ribosome biogenesis, these findings identify TORC1-centered transcriptional networks as a critical hub governing muscle proteostasis during pancreatic cancer–associated cachexia.

We next sought to determine whether TFs associated with TORC1-related processes converge on a common set of downstream target genes. This analysis revealed that multiple distinct TFs converged on 3 shared targets, *Arrdc3*, *Eif4h*, and *Vegfd* (Figure 7B). Notably, *Arrdc3* and *Vegfd* were among the top 50 upregulated genes in cachectic myonuclei compared with myonuclei from control mice (Figure 2C). In addition, eIF4H is a known functional partner of eIF4A within the eIF4F complex and enhances its helicase activity, thereby promoting translational initiation and protein synthesis, linking these convergent targets directly to translational control (14).

To further define the structure of the transcriptional regulatory network, we examined pairwise cooperativity among the prioritized TFs to assess coordinated or divergent regulatory relationships. This analysis identified discrete TF cooperative modules within cachectic myofibers. For example, *Tead1*, *Foxo1*, *Tfdp2*, *Zbtb20*, *Mllt10*, *Mecp2*, and *Arnt* formed a tightly correlated cluster, consistent with shared or convergent regulatory programs. In contrast, other TFs exhibited weak or negative correlations, suggesting divergent transcriptional regulation or potential functional antagonism (Figure 7C). Together, these snRNA-seq–based analyses uncovered previously unrecognized transcriptional coregulatory networks and shared downstream targets that converge on mTOR/TORC1-related processes, highlighting a central role for coordinated transcriptional control of protein synthesis and proteostasis in skeletal muscle during pancreatic cancer cachexia.

### Increased translational initiation and ribosome biogenesis in skeletal muscle of KPC tumor–bearing mice.

**Figure 8:**
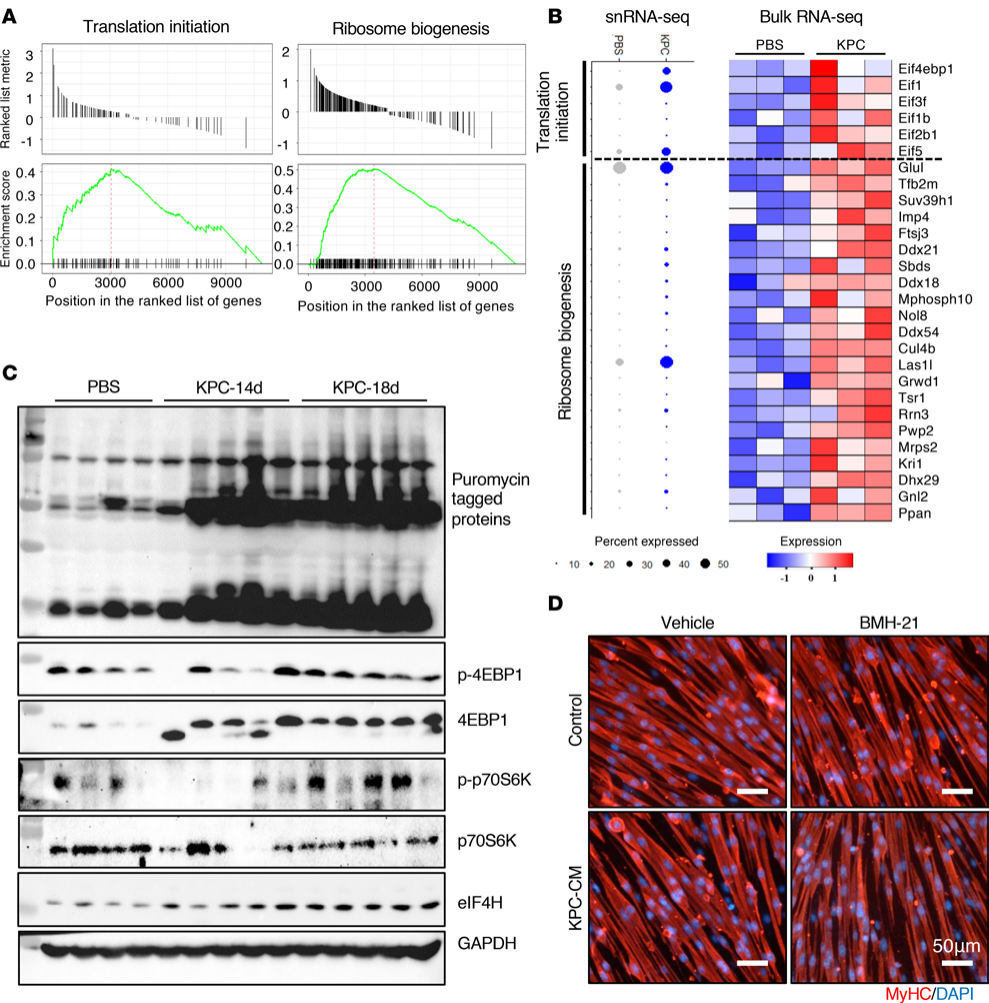
Increased ribosome biogenesis and translation initiation in skeletal muscle of tumor-bearing mice. (**A**) Gene set enrichment analysis of translation initiation and ribosome biogenesis processes in cachectic myonuclear clusters compared with all other nuclei of control mice. (**B**) Dot plots from the snRNA-seq dataset demonstrating the proportion of nuclei expressing translation initiation and ribosome biogenesis genes (left) and heatmap showing corresponding expression in bulk RNA-seq dataset (right). (**C**) Immunoblots showing levels of indicated proteins in TA muscle of control and KPC tumor–bearing mice after 14 and 18 days of inoculation of KPC cells in pancreas. (**D**) Representative photomicrographs of myotube cultures treated with vehicle alone or BMH-21 and incubated with or without KPC cells conditioned medium (KPC-CM).

Skeletal muscle loss during cancer cachexia occurs when protein degradation exceeds protein synthesis (1, 6). Our inferred TF activity analyses indicated that mTORC1 signaling and protein translation pathways are altered in cachectic myonuclei from KPC tumor–bearing mice (Figures 6 and 7). Consistent with this, gene set enrichment analysis (GSEA) of the snRNA-seq dataset revealed significant upregulation of genes whose products are involved in translational initiation and ribosome biogenesis in cachectic myonuclei compared with all myonuclei from control mice (Figure 8A). Similarly, analysis of both snRNA-seq and bulk RNA-seq datasets demonstrated coordinated induction of gene expression of multiple molecules involved in translation initiation and ribosome biogenesis in skeletal muscle of KPC tumor–bearing mice relative to controls (Figure 8B). Independent qPCR analysis confirmed significantly increased mRNA levels of ribosome biogenesis markers, including 18S rRNA, TIF1A, PAF53, Polr1b, UBF, Rpl5, Rpl11, Rps3, and Rps6, in skeletal muscle from KPC tumor–bearing mice compared with controls (Supplemental Figure 6A). To directly assess protein synthesis, we performed surface sensing of translation (SUnSET) assays. Puromycin incorporation was significantly elevated in skeletal muscle of KPC tumor–bearing mice at days 14 and 18 following KPC cell implantation, indicating increased rates of protein synthesis (Figure 8C and Supplemental Figure 6B).

Consistent with enhanced translational initiation, Western blot analyses revealed increased levels of total and phosphorylated 4EBP1, as well as phosphorylated p70S6K, in skeletal muscle from KPC tumor–bearing mice. Moreover, protein levels of eIF4H, one of the convergent transcriptional targets identified in cachectic myonuclei (Figure 7B), were also significantly elevated (Figure 8C and Supplemental Figure 6B). Together, these findings demonstrate that, despite ongoing muscle wasting, skeletal muscle in KPC tumor–bearing mice exhibits increased ribosome biogenesis and translational initiation, suggesting a compensatory or maladaptive activation of protein synthesis pathways during pancreatic cancer–associated cachexia.

We recently reported that KPC cell conditioned media (KPC-CM) induces atrophy in cultured mouse primary myotubes (31). To investigate the functional significance of increased ribosome biogenesis in cachectic muscle, we tested the effect of BMH-21, a small-molecule inhibitor of RNA polymerase I that targets the rate-limiting step of ribosome biogenesis (34), on cultured myotube diameter in control and KPC-CM-treated cultures. Primary mouse myoblasts were differentiated for 48 hours, pretreated with vehicle alone or 250 nM BMH-21 for 2 hours, and then incubated in exhausted differentiation medium (control) or KPC-CM, with or without BMH-21, for an additional 24 hours. Cultures were fixed and immunostained for MyHC, and myotube diameter was measured. Results showed that BMH-21 treatment alone significantly reduced average diameter of cultured myotubes, and this effect was further exacerbated in the presence of KPC-CM (Figure 8D and Supplemental Figure 6C). These results indicate that translational initiation and ribosome biogenesis are upregulated in skeletal muscle during pancreatic cancer cachexia, likely representing an adaptive mechanism that mitigates the severity of muscle wasting.

### Targeted inhibition of TORC1 signaling exacerbates muscle wasting during pancreatic cancer cachexia.

Since we observed an increase in mTORC1 signaling and translation initiation, we next sought to investigate the effect of inhibition of mTOCR1 on the regulation of muscle mass in response to KPC tumor growth. The regulatory-associated protein of mammalian target of rapamycin (Raptor) binds to mTOR and is essential for mTORC1 activity. Previous studies have shown that muscle-specific deletion of Raptor (*Rptor*) leads to a drastic reduction in the protein levels and activity of mTORC1 without affecting muscle mass and function under normal physiological conditions (35, 36). Using inducible muscle-specific *Rptor*-KO mice, we determined whether the inhibition of mTORC1 signaling affects skeletal muscle mass during pancreatic cancer cachexia. Briefly, floxed *Rptor* (henceforth, Rptor^fl/fl^) mice were crossed with HSA-MCM mice to generate Rptor^fl/fl^;HSA-MCM (henceforth, Rptor^mKO^) and littermate Rptor^fl/fl^ mice. Twelve-week-old Rptor^mKO^ mice were treated by i.p. injection of tamoxifen for 4 consecutive days to induce the deletion of Raptor. Littermate Rptor^fl/fl^ mice were also treated with tamoxifen and served as controls. After 2 days of last tamoxifen injection, the mice were injected with PBS alone or 2 × 10^5^ KPC cells into the tail of pancreas and the mice were analyzed on day 21 after inoculation with KPC cells.

**Figure 9:**
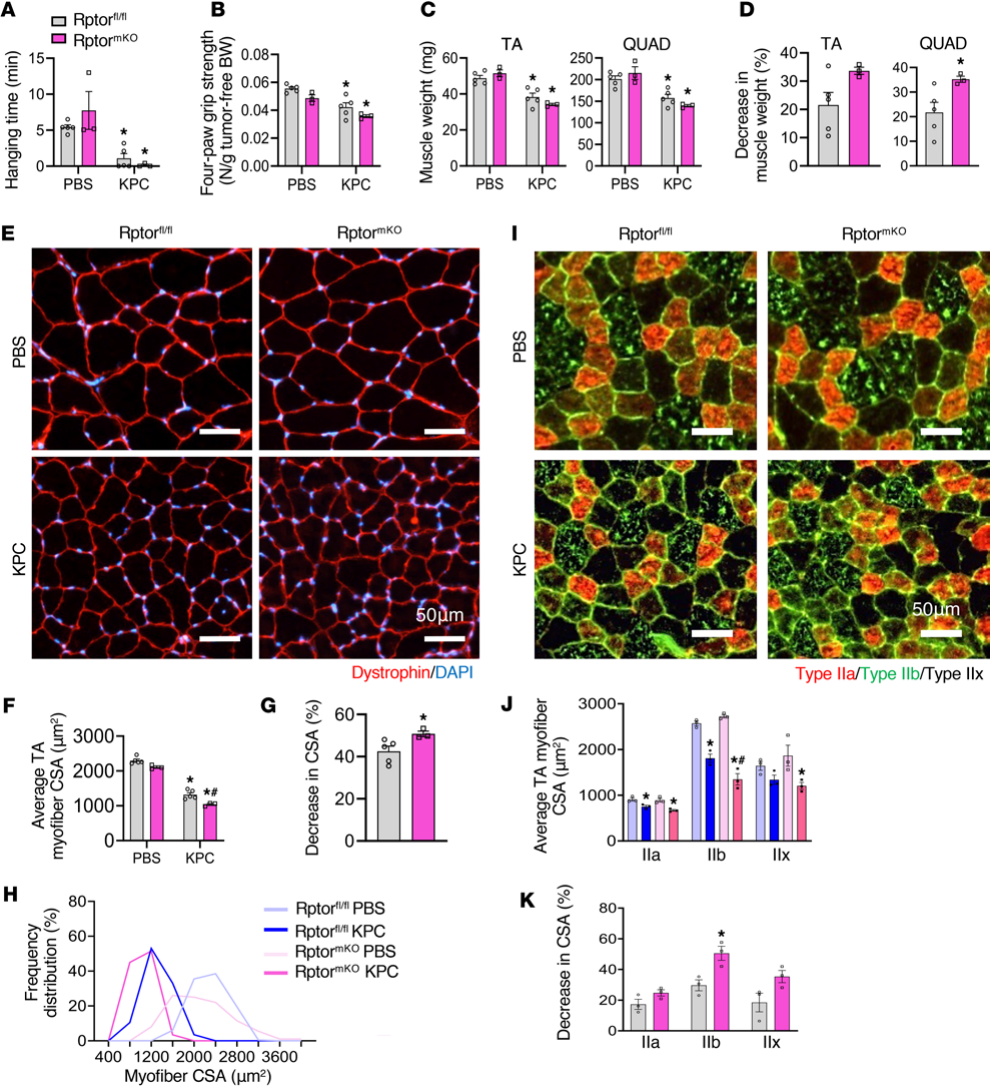
Deletion of Raptor exacerbates muscle loss in response to KPC tumor growth. (**A**–**C**) Wire hanging time (**A**), 4-paw grip strength (**B**), wet muscle weight of tibialis anterior (TA) and quadriceps (QUAD) (**C**) muscle of control and KPC tumor–bearing Rptor^fl/fl^ and Rptor^mko^ mice. (**D**) Tumor-induced decrease in wet weight of TA and QUAD muscle in Rptor^fl/fl^ and Rptor^mKO^ mice. (**E**) Representative images of TA muscle cross-sections after anti-dystrophin and DAPI staining. Scale bar: 50 μm. (**F**–**H**) Myofiber cross-sectional area (CSA) (**F**), decrease in average myofiber CSA (**G**), and myofiber CSA frequency distribution (**H**) in TA muscle of control and KPC tumor–bearing Rptor^fl/fl^ and Rptor^mKO^ mice. (**I**) Representative photomicrographs of TA muscle cross-sections after immunostaining for MyHC isoforms. Scale bar: 50 μm. (**J** and **K**) Myofiber CSA (**J**), and tumor-induced decrease (**K**) in average myofiber CSA in individual muscle type in TA muscle of Rptor^fl/fl^ and Rptor^mKO^ mice. *n* = 3–5 per group. Data are presented as mean ± SEM. *P ≤ 0.05, versus corresponding control mice, ^#^*P* ≤ 0.05, versus KPC-tumor bearing Rptor^fl/fl^ mice (unpaired Student’s *t* test or 2-way ANOVA and Tukey’s multiple-comparison test).

There was a significant reduction in the wire hanging time and 4-paw grip strength normalized by tumor-free body weight in KPC tumor–bearing Rptor^fl/fl^ and Rptor^mKO^ mice compared with corresponding control mice. Although statistical significance was not achieved, there was a trend toward reduced average hanging time and 4-paw grip strength in KPC tumor–bearing Rptor^mKO^ compared with KPC tumor–bearing Rptor^fl/fl^ mice (Figure 9, A and B). There was a significant reduction in the wet weight of TA, quadriceps (QUAD), soleus, and GA muscle of both Rptor^fl/fl^ and Rptor^mKO^ mice in response to KPC tumor growth (Figure 9C and Supplemental Figure 7A). Interestingly, tumor-induced loss in the wet weight of QUAD and GA muscle was significantly higher in Rptor^mKO^ mice compared with Rptor^fl/fl^ mice (Figure 9D and Supplemental Figure 7B). In contrast, there was no significant difference in tumor wet weight between Rptor^fl/fl^ and Rptor^mKO^ mice (Supplemental Figure 7C).

Next, transverse sections of TA muscle were prepared and analyzed by performing H&E staining or immunostaining for dystrophin (Figure 9E and Supplemental Figure 7D), followed by morphometric analysis. The average myofiber CSA was significantly reduced in KPC tumor–bearing Rptor^fl/fl^ and Rptor^mKO^ mice compared with controls. Notably, myofiber CSA was further reduced in KPC tumor–bearing Rptor^mKO^ mice relative to their Rptor^fl/fl^ counterparts (Figure 9, F and G). Consistent with these findings, frequency distribution analysis revealed a shift toward smaller myofibers in Rptor^mKO^ mice compared with Rptor^fl/fl^ mice in response to KPC tumor growth (Figure 9H).

Since Type IIb myofibers are particularly susceptible to cachexia (Figure 4D) and since mTOR signaling genes are selectively upregulated in Type IIb myonuclei (Figure 5A), we next examined fiber-type–specific effects of Raptor deletion. Immunostaining for MyHC isoforms demonstrated that all myofiber types (IIa, IIb, and IIx) exhibited reduced CSA in KPC tumor–bearing Rptor^fl/fl^ and Rptor^mKO^ mice compared with controls (Figure 9, I and J). Importantly, Type IIb myofibers showed the greatest reduction in CSA in Rptor^mKO^ mice relative to Rptor^fl/fl^ mice (Figure 9K), indicating a fiber-type–dependent vulnerability. Efficient deletion of Rptor was confirmed by the reduction in Raptor protein levels in skeletal muscle from Rptor^mKO^ mice compared with Rptor^fl/fl^ mice. Correspondingly, levels of both total mTOR and phosphorylated mTOR were reduced in Rptor^mKO^ mice, irrespective of tumor burden. In addition, a modest increase in ubiquitin-conjugated proteins was observed in skeletal muscle from KPC tumor–bearing Rptor^mKO^ mice compared with Rptor^fl/fl^ mice (Supplemental Figure 7E). Collectively, these findings suggest that targeted inhibition of mTORC1 exacerbates skeletal muscle wasting during pancreatic cancer–associated cachexia.

### Remodeling of inferred intercellular signaling in skeletal muscle in response to KPC tumor growth.

While our previous analyses focused on myofiber-intrinsic transcriptional changes during pancreatic cancer cachexia, nonmyogenic cells in the muscle microenvironment also contribute to muscle wasting. Analysis of nonmyogenic nuclei transcriptome revealed upregulation of genes associated with KEAP1/NFE2L2 signaling, protein catabolism, autophagy, apoptosis, and noncanonical NF-κB pathways, alongside downregulation of genes involved in muscle structure, ECM organization, cytoskeleton, energy metabolism, tube morphogenesis, and blood circulation in MTJ, NMJ, SMC, fibroblast, and endothelial nuclei of KPC tumor–bearing mice (Supplemental Figure 8). These findings indicate that cachexia broadly remodels the transcriptional landscape of nonmyogenic cells as well.

**Figure 10:**
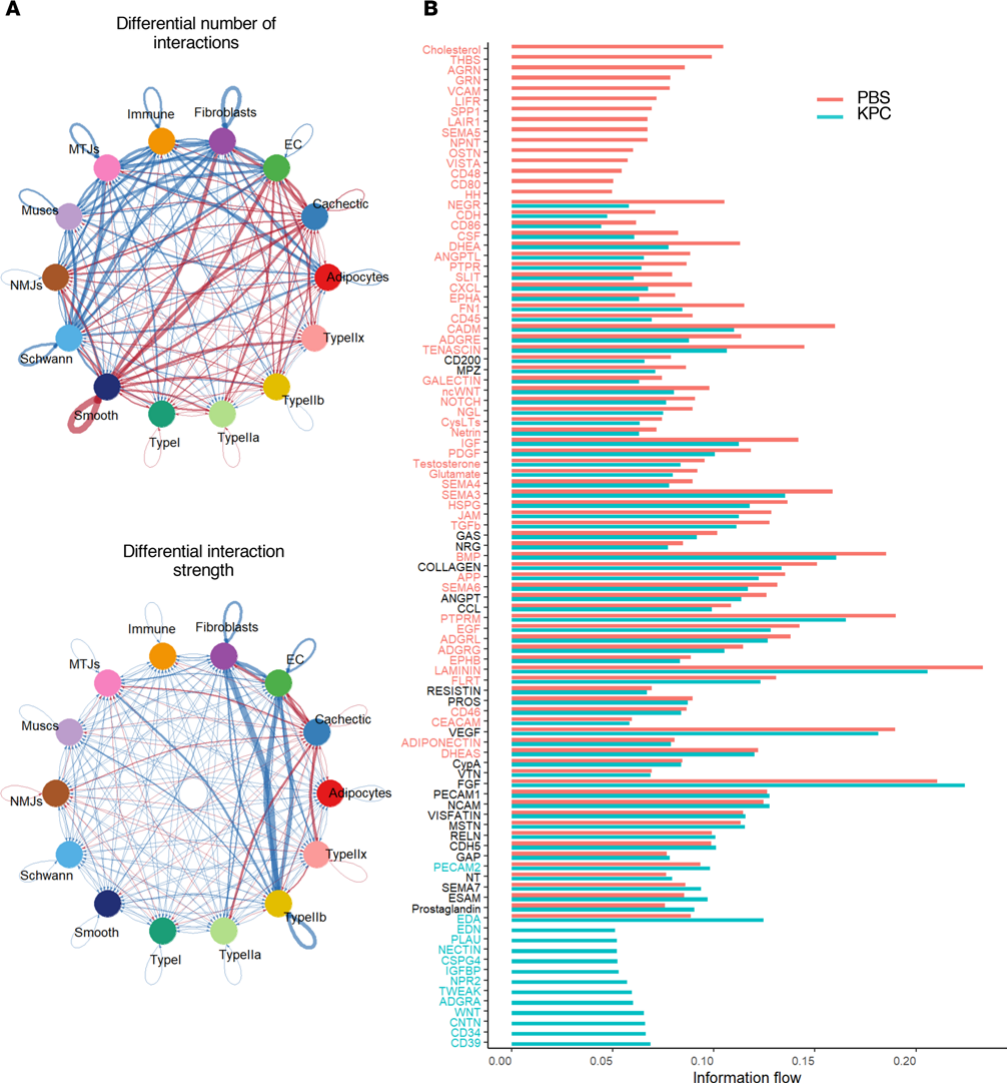
Alterations in inferred intercellular communications in skeletal muscle of KPC tumor–bearing mice. (**A**) Circle plots showing differential number of cellular interactions and strength of interactions between different nuclear clusters of control and KPC tumor–bearing mice. (**B**) Overall information flow comparison of multiple pathways in nuclei of control and KPC tumor–bearing mice.

Next, we investigated the intercellular signaling interactions between nuclear clusters identified in snRNA-seq dataset. For this analysis, we applied CellChat with population weighting to map ligand-receptor–mediated intercellular signaling. Results show that KPC tumor growth induced extensive alterations in both the number and strength of interactions across different muscle and nonmuscle cell types in skeletal muscle (Figure 10A). Further analysis showed that the nuclei in KPC tumor–bearing mice exclusively acquired information flow from various molecules, such as EDA, EDN, PLAU, Nectin, CSPG4, IGFBP, NPR2, TWEAK, ADGRA, WNT, CNTN, CD34, and CD39, which was absent in the nuclear clusters of control mice (Figure 10B and Supplemental Figure 9). In contrast, nuclear clusters of KPC tumor–bearing mice lost information flow from cholesterol, THBS, AGRN, GRN, VCAM, LIFR, SPP1, LAIR1, SEMA5, NPNT, OSTN, VISTA, CD48, CD80, and HH, which were present in the control nuclear clusters (Figure 10B and Supplemental Figure 9). We next investigated how these molecules are affected in different nuclei populations of control versus KPC tumor–bearing mice. This analysis showed that there were significant alterations in signaling from different molecules (e.g., IGF1, Visfatin, MSTN) between nuclear clusters of different cell types in control and KPC tumor–bearing mice (Figure 10B and Supplemental Figure 10A). Moreover, CD39 signaling was present in EC and smooth muscle nuclei, whereas CD34 was present in only EC of tumor-bearing mice. CNTN and Wnt were present in almost all types of nuclei of tumor-bearing mice, but not in control mice. TWEAK, a potent muscle wasting cytokine (31, 32), was present in cachectic, MTJ, smooth muscle, and Type IIa and Type IIb nuclear clusters (Supplemental Figure 10B and Supplemental Figure 11A). We also found that strength of information flow from several other molecules implicated in muscle wasting, such as EDA, Prostaglandin, CD46, NGL, Notch, Testosterone, CCL, MSTN, Visfatin, fibronectin, ANGPT, TGF-β, FLRT, BMP, and FGF, was considerably increased in cachectic nuclei of KPC tumor–bearing mice, even though some of these signals were present in Type II myofibers of both control and tumor-bearing mice (Supplemental Figure 11A). We performed further analyses to identify the ligand/receptor enrichment in our snRNA-seq dataset. In control muscle, enrichment was dominated by Col4a3, Mpz, Lama2, Tnxb, and Col4a1, consistent with a structural extracellular matrix (ECM) and NMJ supportive environment. In contrast, KPC muscle shifted toward Fn1, Col4a1, Col4a2, and Vegfa reflecting ECM remodeling and altered vascular-endocrine communication (Supplemental Figure 11B). Together, these integrated metrics demonstrate reprogramming of intercellular signaling in a directional and pathway-specific manner, supporting catabolic alterations such as ECM remodeling, inflammation, and neuromuscular degeneration, in skeletal muscle during pancreatic cancer cachexia.

## Discussion

Cachexia is a major catabolic condition during malignant tumor progression involving the loss of skeletal muscle mass with or without concomitant fat loss (8). However, the etiology of cancer-induced cachexia remains less understood. In the present study, we have used snRNA-seq and bulk RNA-seq approaches to investigate the molecular changes that occur in different cell types present in skeletal muscle microenvironment in the KPC mouse model of pancreatic cancer cachexia. We have identified a unique myonuclei population that we termed as cachectic cluster, which expresses the molecular signature of muscle atrophy. Our analysis also demonstrates that the transcriptional regulation, specifically in the Type II myofibers, induces transition of myonuclei toward the cachectic state. Furthermore, our results demonstrate that several common and distinct transcriptional changes occur in each myofiber types in response to tumor growth (Figures 4 and 5).

UPS and autophagy are the major mechanisms driving muscle proteolysis in various catabolic conditions, including cancer-induced cachexia (3, 6, 37). Some studies suggest that the Type IIb myofibers undergo atrophy at faster rate compared with other types of myofibers in various atrophying conditions, including cancer cachexia (38, 39). Our analysis of the snRNA-seq dataset showed that molecular signatures of both UPS and autophagy are highly upregulated in cachectic myonuclei as well as Type IIb and IIx myonuclear clusters, suggesting that glycolytic myofibers are more susceptible to wasting compared with oxidative myofibers during cancer cachexia (Figures 2 and 4). Our analysis further showed that the gene expression of many other molecules (e.g., Mt1/Mt2, Vegfd, Elk4, Retreg1, Arrdc3) is highly induced in cachectic myonuclei. Future studies will characterize the role of these proteins in the etiology of muscle wasting during cancer cachexia.

Mitochondrial dysfunction is a well-established and common pathological feature in the skeletal muscle during cancer cachexia (3, 40). Tumor-derived factors and the host’s inflammatory response trigger several overlapping mechanisms that inhibit mitochondrial biogenesis and cause mitochondrial damage and impaired energy metabolism (40). Damaged mitochondria can also cause oxidative stress, which can independently activate catabolic pathways in skeletal muscle. Indeed, we recently reported increase in fatty acid accumulation and metabolism and oxidative stress in skeletal muscle of KPC tumor–bearing mice (26). Moreover, another study showed that cAMP/PKA/CREB signaling is a central mechanism contributing to mitochondrial dysfunction in skeletal muscle in animal models of cancer cachexia. Furthermore, transcriptome analysis suggested that this pathway is repressed in skeletal muscle of patients with PDAC (41, 42). Our analysis of snRNA-seq dataset revealed that the gene expression of many molecules related to OXPHOS is diminished in cachectic myonuclei compared with controls (Figure 3). These findings suggest that reduced mitochondrial content and function is one of the important pathological features of atrophying skeletal muscle during pancreatic cancer cachexia.

Recently, it was also reported that the vascular supply is diminished in skeletal muscle during cancer induced cachexia (12). The angiogenic program in skeletal muscle is governed not only by the endothelial and vascular smooth muscle cells, but also by the factors produced by myofibers (43). Our study provides evidence that the gene expression of several molecules that support angiogenesis is diminished in all myonuclear clusters except in Type I myonuclei. Bulk RNA-seq and histological analysis further confirmed that gene expression of various angiogenesis-related molecules and vascular supply is diminished in skeletal muscle of KPC tumor–bearing mice (Figure 3).

Our bioinformatics analysis demonstrates that the activity of specific TFs and the expression of their target genes is significantly upregulated in the cachectic myonuclear clusters of KPC tumor–bearing mice compared with myonuclei of control mice. While the role of some of these TFs (e.g., Foxo1) in the regulation of muscle mass has been elucidated previously (44, 45), we identified several other TFs that are highly upregulated and play a coregulatory role in cachectic myonuclei but their specific role in cancer-induced muscle wasting has not yet been investigated (Figures 6 and 7). Intriguingly, our analysis showed that mTORC1 signaling, ribosome biogenesis, and translation initiation are some of the major processes associated with the activated TFs in cachectic myonuclei of KPC tumor–bearing mice (Figure 7). A previous study has shown that ribosome biogenesis and protein synthesis are diminished in skeletal muscle of a mouse model of ovarian cancer-induced cachexia (46). In addition, there are other reports that suggest significant reduction, no change, or upregulation of protein synthesis in skeletal muscle of animal models of cancer cachexia (13, 30). Consistent with the snRNA-seq analysis, our independent biochemical analysis showed that the markers of ribosome biogenesis, translation initiation, and protein synthesis are significantly upregulated in skeletal muscle of KPC tumor–bearing mice (Figure 8 and Supplemental Figure 6A). While the upregulation of protein synthesis and ribosome biogenesis may represent a compensatory response to counteract excessive proteolysis, apparently, it is not sufficient to prevent muscle wasting during pancreatic cancer cachexia.

Activation of the mTORC1 complex is a central mechanism that enhances protein synthesis in response to mechanical loading, nutrient availability, and other anabolic stimuli (45, 47). However, accumulating evidence suggests that both acute activation and suppression of mTORC1 disrupt autophagy and impair skeletal muscle homeostasis, ultimately leading to muscle wasting and myopathy (48, 49). Forced activation of mTORC1 exacerbates denervation-induced muscle atrophy by inhibiting Akt kinase through a negative-feedback mechanism (50). Moreover, mTORC1 activation has been implicated in age-related muscle loss, potentially via enhanced phosphorylation of the STAT3 TF (17). Interestingly, forced overexpression of follistatin, which antagonizes myostatin and Activin A signaling, promotes protein synthesis and skeletal muscle hypertrophy through activation of the Akt/mTORC1/p70S6K pathway (51), suggesting that mTORC1-mediated signaling can either promote or deteriorate muscle mass in a context-dependent manner.

Our results demonstrate that the mTORC1-mediated signaling prevents the excessive loss of skeletal muscle mass during pancreatic cancer cachexia (Figure 9). In addition, our experimentation provides insights into the fiber-type–specific role of mTORC1 signaling in pancreatic cancer cachexia. The mTORC1-mediated adaptive response was observed selectively in Type IIb myofibers (Figure 5A), supported by the findings that inactivation of mTORC1 results in a profound decrease in the size of Type IIb myofibers compared with other muscle fiber types in response to KPC tumor growth (Figure 9K). These findings are also supported by a previously published report, which demonstrates that forced activation of Akt/mTORC1 signaling inhibits skeletal muscle wasting in other models of cancer cachexia (52). Altogether, our results provide initial evidence that skeletal muscle undergoes transcriptional reprograming to enhance translation capacity and efficiency to prevent extreme muscle wasting during pancreatic cancer cachexia.

In addition to myofibers, skeletal muscle contains many other cell types which may also play important roles in the regulation of muscle mass in tumor-bearing hosts. Our snRNA-seq analysis demonstrates that tumor growth affects the transcriptome of other cell types, such as EC, smooth muscle, and fibroblasts present in skeletal muscle microenvironment. Moreover, cell-cell communication is altered in skeletal muscle of tumor-bearing mice compared with controls (Figure 10). While the signaling from IGF1 is diminished, there is an increase in the signaling from catabolic molecules, such as TWEAK, EDA, and myostatin in cachectic myonuclei. Interestingly, some of the catabolic molecules are produced predominantly by nonmuscle cells which then act on myofibers, which express cell surface receptors for them (Supplemental Figure 9–11). This selective rewiring in muscle microenvironment may explain fiber-type vulnerability and establishes a cachectic microenvironment that promotes progressive myofiber loss.

Our findings from the KPC model of cancer cachexia show concordance with a recently published study using the KIC model of pancreatic cancer cachexia (11). Notably, we observed similar upregulation of multiple genes, including canonical atrophy markers such as MAFbx and MuRF1, as well as other genes including *Vegfd*, *Tfdp2*, *Eda2r*, and *Eif4ebp1*. These shared transcriptional changes suggest a conserved transcriptional activation of a set of genes whose products directly or indirectly contribute to skeletal muscle wasting across distinct models of PDAC-induced cachexia. Our snRNA-seq analysis also reveals model-specific features. For example, we observed 2 cachexia-associated myonuclear clusters exhibiting largely similar transcriptional profiles with some modest differences (Supplemental Figure 1B). In contrast, Zhang et al. study showed the presence of distinct “denervated” and “catabolic” myonuclear clusters, indicative of denervation and proteolysis as major contributors of muscle wasting in the KIC model (11). While our cell-cell communication analysis revealed enrichment of myostatin signaling between Type IIb and cachectic myonuclei, the denervation phenotype was not readily observed in our KPC model of pancreatic cancer cachexia. This discrepancy may reflect model-specific transcriptomic adaptations, suggesting that the relative contributions of conserved pathogenic mechanisms vary across experimental models of pancreatic cancer cachexia.

While, in this study, we have used mouse model of cancer cachexia, our dataset shows overlapping features with transcriptional changes observed in skeletal muscle of patients with PDAC. For instance, ECM remodeling and mitochondrial dysfunction are shared transcriptomic signatures between the KPC model used in this study and cachectic patient datasets (21, 41, 53).

In addition, our observation that glycolytic muscle fibers are more susceptible to atrophy is consistent with findings in skeletal muscle from patients with pancreatic cancer (21). However, like most mouse models of PDAC-associated cachexia, our study also demonstrates increased activation of E3 ubiquitin ligases, a response that does not fully align with transcript levels reported in muscle biopsies from cachectic patients (21, 53). These observations suggest that, even though many phenotypic features of muscle wasting are shared, important differences remain between the skeletal muscle transcriptomes of patients with PDAC and commonly used mouse models of cancer cachexia.

While our study has provided important insights into the molecular mechanisms underlying muscle wasting during pancreatic cancer cachexia, it also has several limitations. Notably, the snRNA-seq analysis was conducted at a single time point in both control and tumor-bearing mice. Future studies examining gene expression dynamics across different stages of tumor progression would offer a more comprehensive understanding of disease development. Additionally, it remains to be determined whether similar transcriptional changes also occur in skeletal muscle across other models of cancer cachexia. Furthermore, key findings from the snRNA-seq analysis, particularly the involvement of specific TFs, warrant further investigation using genetic mouse models. Despite these limitations, our study identifies previously unknown mechanisms and potential molecular targets relevant to pancreatic cancer cachexia.

## Methods

### Sex as a biological variable.

All experiments involving animals were conducted using male mice at 11–12 weeks of age because male animals exhibit cachexia in response to pancreatic cancer. It is unknown whether similar findings will also be obtained for female mice.

### Animals.

C57BL/6, floxed Raptor (*Rptor^fl/fl^*; Strain: B6.Cg-Rptor^tm1.1Dmsa^/J), and HSA-MCM (Strain: B6.Cg-Tg[ACTA1-cre/Esr1*]2Kesr/J) mice were purchased from Jackson Laboratory. *Raptor^fl/fl^* mice were crossed with HSA-MCM mice to generate muscle-specific Raptor-KO (*Rptor^mKO^*) and littermate control (*Rptor1^fl/fl^*) mice. All mice were in the C57BL/6 background, and their genotype was determined by PCR from tail DNA. We employed KPC orthotopic mouse model of pancreatic cancer cachexia (20, 54). KPC cells, as described (54), were provided by Elizabeth Jaffee (Johns Hopkins University). Briefly, KPC cells (2 × 10^5^ cells in 20 μL PBS) were injected into the tail of the pancreas of 12-week-old WT or *Rptor^fl/fl^* and *Rptor^mKO^* mice. Control mice received an injection of PBS alone. The mice were weighed weekly and euthanized on day 14–21 after the injection of KPC cells. All the animals were handled according to the approved institutional animal care and use committee (IACUC; protocol no. PROTO201900043) of the University of Houston. All surgeries were performed under anesthesia, and every effort was made to minimize suffering.

### Cell culture and immunostaining.

Culturing of primary myoblasts and their differentiation into myotubes and immunostaining protocol is described in Supplemental Methods.

### Grip strength test.

Four-paw grip strength of mice was measured using a digital grip strength meter (Columbus Instruments), as previously described (55).

### Histology and morphometric analysis.

The details about muscle histology and morphometric analysis are provided in the Supplemental Methods.

### Western blot.

Western blot analysis was performed following exactly same procedure as previously described (56). Primary and secondary antibodies used in western blots are described in Supplemental Table 1. Uncropped gel images are provided in Supplemental Figure 12.

### RNA extraction and qPCR.

RNA isolation and qPCR were performed following a protocol as previous described (56). The sequence of the primers is described in Supplemental Table 2.

### Bulk RNA-seq analysis.

The bulk RNA-Seq was performed following a method as described (26) and detailed in the Supplemental Methods. All the raw data files can be found on the NCBI SRA repository using the accession code PRJNA1255232.

### snRNA-seq.

Freshly isolated GA muscle tissues were processed for the isolation of nuclei. The nuclei were pooled from 5 mice in each group. snRNA-seq libraries were generated using Chromium Single Cell 3’ v3 kit (10X Genomics) according to the manufacturers protocol. Sequencing was performed on an Illumina NovaSeqXPlus system. The sample demultiplexing was performed based on 10 bp i5 and i7 sample index reads to generate Read1 and Read2 paired-end reads. The Cell Ranger Software Suite (10X Genomics, v7.0.0) was used for data demultiplexing, transcriptome alignment, and UMI counting. Raw data are stored in FASTQ (fq) format files, which contain sequences of reads and corresponding base quality. The mouse genome (GRCm38), version M23 (Ensembl 98), was used as reference for read alignments and gene counting with cellranger count. The Seurat R package was further utilized to achieve data quality control. All the raw data files can be found on the NCBI SRA repository using the accession code PRJNA1330413.

### snRNA-seq downstream analysis.

The protocol used for the analysis of snRNA-seq dataset is described in the Supplemental Methods.

### Louvain community detection and trajectory analysis.

Pseudotemporal dynamics were reconstructed using Monocle3 (v1.3.7) (57). Seurat objects from KPC and PBS samples were converted to Monocle3 cell_data_set objects via as.cell_data_set(). Only myonuclei clusters (e.g., Type IIa, IIb, IIx, and cachectic myonuclei) were retained based on prior annotations. Dimensionality reduction was performed using 50 principal components (num_dim = 50), and UMAP coordinates from Seurat were directly transferred to ensure consistency in embedding across analyses. Cells were clustered in Monocle3, and trajectories were learned using the learn_graph() function. The trajectory was mapped by designating the Type IIa and Type IIb myonuclei as root (i.e., origin) and Cachectic nuclei cluster as terminal node. Graph-based differential expression testing was performed using graph_test() with the principal graph or kNN graph as the neighborhood structure (neighbor_graph = “principal_graph” or “knn”). Genes with FDR-adjusted *q* < 0.05 were retained. We applied Louvain community detection analysis to identify gene modules via find_gene_modules() using resolution parameters ranging from 1 × 10^–6^ to 1 × 10^–1^ to capture modules at multiple scales. Module expression was aggregated across cell partitions using aggregate_gene_expression(), and module–partition expression heatmaps were visualized using Ward’s method for clustering.

### Comparative intercellular communication analysis.

We profiled condition-resolved signaling with CellChat (v2.1.2) as described (58) on a Seurat object split by group labels (PBS, KPC). The details of this analysis are provided in Supplemental Methods.

### SCENIC and transcription network analysis.

This analysis has been described in the Supplemental Methods.

### Augur-based perturbation sensitivity.

Determination of cell-state sensitivity to cachexia was performed using machine learning-based Augur method on a Seurat object (59). Briefly, Augur method quantifies how strongly each cell population responds transcriptionally to a perturbation (here, control versus KPC) by training a classifier (random forest) to distinguish cells from control versus cachectic conditions using repeated subsampling (50 iterations; 20 cells per class) with 3-fold cross-validation. Area under ROC curve (AUC) values were averaged across subsamples to rank perturbation sensitivity. Cell types with higher Augur scores were those in which cachexia induced stronger transcriptional divergence from baseline, which we refer to as increased “sensitivity to cachexia.” Analyses were performed in R (Seurat v5.3.0, Augur v1.0.3).

### Pathway module scoring and visualization.

We quantified pathway-level activity per nucleus using curated gene sets for autophagy, the UPS, OXPHOS, and angiogenesis. Metadata were harmonized by renaming orig.ident to KPC or PBS, and coarse cell types were derived from Seurat clusters (e.g., Type I, Type IIa/IIb/IIx, cachectic myonuclei). For each gene set, per-cell module scores were computed with Seurat AddModuleScore on the RNA assay (60). Gene-level expression was summarized with DotPlot grouped by cell type and split by condition (KPC versus PBS). Pathway activity distributions were visualized with VlnPlot for the corresponding module score (split by condition across cell types).

### Statistics.

All wet-lab data are presented as mean ± SEM. Statistical analyses were performed using GraphPad Prism 10.0. Comparisons between 2 groups were conducted using 2-tailed unpaired Student’s *t* test. For experiments involving multiple comparisons, 2-way ANOVA followed by Tukey’s post hoc test was applied. *P* ≤ 0.05 was considered statistically significant. Additional statistical details, including exact *n*, are provided in the corresponding figure legends.

### Study approval.

All animal procedures were conducted in strict accordance with the institutional guidelines and were approved by the IACUC and Institutional Biosafety Committee of the University of Houston (PROTO201900043).

### Data availability.

All relevant raw data related to this manuscript are available in the Supporting Data Values file. Raw data files for RNA-seq experiments can be found in the NCBI SRA repository using the accession code PRJNA1255232 and PRJNA1330413.

## Author contributions

AK designed the work. BX, ASJ, MTDS, and SL performed the experiments and analyzed the results. BX and ASJ wrote the first draft of the manuscript. AK and other authors edited and finalized the manuscript.

## Funding support

This work is the result of NIH funding, in whole or in part, and is subject to the NIH Public Access Policy. Through acceptance of this federal funding, the NIH has been given a right to make the work publicly available in PubMed Central.

- NIH/National Institute of Arthritis and Musculoskeletal and Skin Diseases (AR081487)
- NIH/National Cancer Institute (CA294365)

## Supplementary Material

Supplemental data

Unedited blot and gel images

Supporting data values

## Version 1 01/27/2026

In-Press Preview

## Version 2 03/23/2026

Electronic publication
