# Supplementary material for: Fiber-type vulnerability and proteostasis reprogramming in skeletal muscle during pancreatic cancer cachexia
Source: JCI Insight. 2026 Jan 27;11(6):e200396. doi: 10.1172/jci.insight.200396 (PMC13043100; doi:10.1172/jci.insight.200396)
Supplement: Supplemental data [file jciinsight-11-200396-s346.pdf]

Fiber-type vulnerability and proteostasis reprogramming in skeletal muscle during pancreatic cancer cachexia

Bowen Xu, Aniket S. Joshi, Meiricris Tomaz da Silva, Silin Liu, and Ashok Kumar

This file contains:

Supplemental Methods

Supplemental Figures (S1-S12) and legends

Tables S1 and S2

Supplemental references

Supplemental Methods

Cell culture and immunostaining. KPC cells were kindly provided by Dr. Elizabeth Jaffee (Johns Hopkins University, Baltimore, MD) and cultured in RPMI-1640 medium supplemented with 10% fetal bovine serum (FBS). To prepare KPC cell-conditioned medium (KPC-CM), cells were grown to confluency, then incubated for 24 hours in differentiation medium (DM; DMEM supplemented with 2% horse serum). The supernatant was collected, clarified by centrifugation, and filtered through a sterile 0.22 μm syringe filter. For myotube atrophy experiments, KPC-CM was diluted 1:4 in fresh DM.

Primary myoblasts were isolated from hindlimb muscle of C57BL/6 mice as described (1). For myotube formation, primary myoblasts were incubated in DM for 48 h. Myotubes were treated with vehicle alone (DMSO) or BMH-21 for 2h followed by addition of DM (control) or KPC-CM with or without BMH-21 and incubated for additional 24 h. For immunostaining, the cultures were fixed with 4% PFA in PBS for 15 min at room temperature and permeabilized with 0.1% Triton X-100 in PBS for 10 min. Cells were blocked with 2% bovine serum albumin in PBS and incubated with mouse-anti-MyHC (clone MF20, DSHB, Iowa City, Iowa) overnight at 4 °C. The cells were then washed with PBS and incubated with a secondary antibody at room temperature for 1 h. Nuclei were counterstained with DAPI for 3 min. Average myotube diameter was calculated by measuring diameter of 100-120 MyHC^{+}-myotubes per group using the NIH ImageJ software. For consistency, diameters were measured at the midpoint along with the length of the MyHC^{+} myotubes.

Histology and morphometric analysis. Individual TA and soleus muscles were isolated from mice, snap-frozen in liquid nitrogen, and sectioned with a microtome cryostat. For the

assessment of muscle morphology, 8-μm-thick transverse sections of TA muscle were stained with hematoxylin and eosin (H&E) dye. Muscle sections were also processed for immunostaining for laminin protein to mark the boundaries of myofibers. Briefly, frozen transverse sections of TA or soleus muscle were fixed in either acetone or 4% paraformaldehyde (PFA) in PBS, followed by blocking with 1% bovine serum albumin (BSA) in PBS for 1 h at room temperature. Sections were incubated overnight at 4°C under humidified conditions with primary antibodies against Type I, Type IIa, and Type IIb myosin heavy chains (1:100; DSHB, University of Iowa, Iowa City, IA), laminin (1:500; Sigma Chemical Co.), or laminin and CD31 protein diluted in blocking solution. After brief washes in PBS, sections were incubated with Alexa Fluor 350-, 488-, or 568-conjugated secondary antibodies (1:500; Invitrogen) for 1 h at room temperature, followed by three 5-min washes in PBS. Nuclei were counterstained with DAPI. Slides were mounted with fluorescence mounting medium (Vector Laboratories) and imaged at room temperature using a Nikon Eclipse Ti-2E inverted microscope equipped with a Digital Sight DS-Fi3 camera and Nikon NIS-Elements AR software. Image levels were uniformly adjusted in Adobe Photoshop CS6 (Adobe). For morphometric analysis, myofiber cross-sectional area (CSA) was analyzed in anti-laminin-stained muscle sections using ImageJ software (NIH, Bethesda, MD). For each muscle, the CSA distribution was determined by analyzing approximately 200 myofibers. Similarly, we quantified the number of $CD31^+$ cells per unit area in soleus muscle section using ImageJ software.

Surface sensing of translation (SUnSET) assay. The rate of protein synthesis in skeletal muscle was measured following a protocol as described (2, 3). In brief, Mice were administered puromycin by intraperitoneal injection (0.04 μmol per gram of body weight). Exactly after 30

minutes, the mice were euthanized, and hindlimb muscles were excised and snap-frozen. Protein extracts were then prepared from muscle tissues and newly synthesized proteins were detected by immunoblotting using an anti-puromycin primary antibody.

Bulk RNA-seq analysis. Total RNA from GA muscle of control and KPC tumor-bearing mice was extracted using TRIzol reagent (Thermo Fisher Scientific) using the RNeasy Mini Kit (Qiagen, Valencia, CA, USA) according to the manufacturer's protocols. The mRNA-seq library was prepared using poly (A)-tailed enriched mRNA at the UT Cancer Genomics Center using the KAPA mRNA HyperPrep Kit protocol (KK8581, Roche, Holding AG, Switzerland) and KAPA Unique Dual-indexed Adapter kit (KK8727, Roche). The Illumina NextSeq550 was used to produce 75 base paired-end mRNA-seq data at an average read depth of ~38 M reads/sample. RNA-seq fastq data were processed using CLC Genomics Workbench 20 (Qiagen). Illumina sequencing adapters were trimmed, and reads were aligned to the mouse reference genome Refseq GRCm39.105 from the Biomedical Genomics Analysis Plugin 20.0.1 (Qiagen). Normalization of RNA-seq data was performed using a trimmed mean of M values. Genes with $Log2FC \geq 0.25$ and $p\text{-value} < 0.05$ were assigned as differentially expressed genes (DEGs) and represented in volcano plot using ggplot function in R software (v 4.2.2). Pathway enrichment analysis was performed using the Metascape Gene Annotation and Analysis tool (metascape.org). Heatmaps were generated by using heatmap.2 function using z-scores calculated based on transcripts per million (TPM) values. Genes involved in specific pathways were manually selected for heatmap expression plots. All the raw data files can be found on the NCBI SRA repository using the accession code PRJNA1255232.

snRNA-seq downstream analysis. Cell calling was performed using the EmptyDrops algorithm, which identifies cell-associated barcodes based on unique molecular identifier (UMI) counts and RNA content. Quality control was conducted using Seurat, and nuclei were excluded if they met any of the following criteria: fewer than 200 genes detected, genes with non-zero counts in fewer than 3 nuclei, more than 8000 detected genes (to exclude potential multiplets), mitochondrial gene content greater than 50%, or hemoglobin-related gene content greater than 5%. After filtering, gene expression matrices were normalized by total UMI counts per nucleus, scaled by a sample-specific median UMI factor, and log-transformed using the log1p method. Feature selection was performed to remove uninformative genes and identify biologically relevant signals. Highly variable genes (HVGs) were identified using the FindVariableGenes function in Seurat with the vst method, and the top 3000 HVGs were selected for each sample. Principal component analysis (PCA) was conducted using these HVGs to reduce dimensionality and capture major axes of transcriptomic variation. The resulting principal components were used for unsupervised clustering and identification of cell subpopulations. For visualization, dimensionality was further reduced to two dimensions using t-distributed stochastic neighbor embedding (t-SNE) and uniform manifold approximation and projection (UMAP).

Comparative Intercellular Communication Analysis. We profiled condition-resolved signaling with CellChat (v2.1.2) as described (4) on a Seurat object split by group labels (PBS, KPC). Cell types were curated by renaming Seurat clusters and stored as meta.data$new_clusters (e.g., Type IIb, Type IIx, Type IIa, Type I, cachectic myonuclei, fibroblasts, endothelial cells, Schwann cells, MTJ/NMJ nuclei, immune, adipocytes, smooth muscle, MuSCs). For each condition, we used the RNA assay data slot (log-normalized counts), retained genes detected in

≥10 cellsand harmonized the LR database by enforcing unique Symbol rownames. CellChat objects were created followed by subsetData(), identifyOverExpressedGenes(), and identifyOverExpressedInteractions(). Communication probabilities were estimated with computeCommunProb (raw.use = TRUE, population.size = TRUE, type = "truncatedMean"), aggregated to pathways via computeCommunProbPathway() and aggregateNet(), and then compared conditions after merging objects with mergeCellChat(). We contrasted the number and overall strength of interactions using compareInteractions() and visualized rewiring with circle plots, global heatmaps, and netVisual_diffInteraction(). Sender or receiver roles were quantified with netAnalysis_computeCentrality() and netAnalysis_signalingRole_scatter(), and condition-specific changes were profiled for myonuclei using netAnalysis_signalingChanges_scatter(). Pathway architecture was evaluated by computing functional and structural similarities with computeNetSimilarityPairwise(), embedding joint manifolds via netEmbedding(), clustering with netClustering(), and summarizing distances/rankings using rankSimilarity() and rankNet(measure = "weight"). Differential LR analysis on the merged object used identifyOverExpressedGenes(thresh.pc = 0.1, thresh.fc = 0.05, thresh.p = 0.05), mapping DE results back to communications with netMappingDEG() and extracting LR sets via subsetCommunication() (ligand.logFC thresholds: +0.05 for up-regulated in KPC, −0.05 for down-regulated). Regulated LR programs were visualized with bubble plots, chord diagrams, and word clouds, and gene expression distributions were compared across groups with plotGeneExpression.

SCENIC and transcription network analysis. Single-nucleus RNA-seq data were processed in Seurat (v5.3.0) (5). Nuclei passing basic QC (same as above) were retained, log-normalized, and

highly variable genes were identified with FindVariableFeatures (vst). For TF program inference we ran pySCENIC (v0.12.1) on Linux/WSL2 in three steps: (i) GRN inference with pyscenic grn using GRNBoost2 and a curated mouse TF list (TF_symbols.txt supplied with pySCENIC resources); (ii) motif enrichment/pruning with pyscenic ctx against the mm10 cisTarget ranking databases (v10_clust; 10 kb up/10 kb down and 500 bp up/100 bp down) and the v10nr motif annotations table, combining promoter-proximal and distal evidence; and (iii) AUCell scoring with pyscenic aucell to produce per-cell regulon activity in a .loom. SCENIC outputs were imported into R and the AUCell matrix (RegulonsAUC × CellID) was added as a Seurat assay (RegAUC). Loom Cell IDs were aligned to Seurat barcodes, and basic coverage checks confirmed that TFs/targets from the GRN were present in the expression matrix. Differential regulon activity between KPC and PBS groups was tested on RegAUC with two-sided Wilcoxon tests and Benjamini–Hochberg (BH) correction (logfc.threshold = 0 to retain subtle activity shifts; downstream filters control specificity). Unless noted, these comparisons were performed across all myofiber nuclei; cluster-stratified analyses were additionally performed for cachexia-associated clusters (e.g., clusters 2 and 14) where indicated. "Triple-evidence" TFs were defined as those that satisfied all three criteria: (i) significant up-regulation of the TF's regulon activity in cachectic myofibers (RegAUC, BH FDR < 0.05); (ii) overlap between SCENIC-inferred high-confidence targets and a high-confidence cachexia DEG set from RNA (Wilcoxon, BH FDR < 0.05, avg_log2FC > 0; computed within clusters 2/14 for atrophy-focused contrasts); and (iii) up-regulation of the TF's own transcript (RNA, BH FDR < 0.05). For visualization, regulon AUCs were row-wise z-scored and hierarchically clustered (ComplexHeatmap); UMAP feature maps were drawn for key regulons (RNA-based PCA/UMAP for display), and AUCell thresholds were estimated per regulon (AUCell_exploreThresholds) to binarize activity and compute the fraction

of active cells by cluster/condition. TF cooperativity within cachexia-associated clusters was assessed using Pearson correlations of RegAUC followed by Ward.D2 clustering to define modules. High-confidence targets of triple-evidence TFs underwent GO Biological Process enrichment (clusterProfiler enrichGO, mouse OrgDb; BH-adjusted $q \leq 0.05$). An enrichment map linked terms by Jaccard overlap ≥ 0.2, followed by Louvain community detection and calculation of degree, betweenness, and closeness centralities; terms explicitly referring to TORC1/mTOR were annotated as "core mTOR", and translation/ribosome terms as "indirect translation/ribosome". To connect TFs to functions, a Sankey diagram linked triple-evidence TFs to enriched GO terms with ribbon width proportional to the number of high-confidence targets contributing to each term. A directed TF-TF network was generated by treating SCENIC targets that are TFs as downstream nodes (self-loops removed) and plotted with a force-directed layout (ggraph/igraph; node size = out-degree). Unless stated otherwise, tests were two-sided with BH correction.

Pathway module scoring and visualization. We quantified pathway-level activity per nucleus using curated gene sets for autophagy, the ubiquitin–proteasome system (UPS), oxidative phosphorylation (OXPHOS) and angiogenesis. Metadata were harmonized by renaming orig.ident to KPC or PBS, and coarse cell types were derived from Seurat clusters (e.g., Type I, Type IIa/IIb/IIx, cachectic myonuclei). For each gene set, per-cell module scores were computed with Seurat AddModuleScore on the RNA assay (6). Gene-level expression was summarized with DotPlot grouped by cell type and split by condition (KPC vs PBS). Pathway activity distributions were visualized with VlnPlot for the corresponding module score (split by condition across cell types).

Supplemental Figures and Legends

FIGURE S1

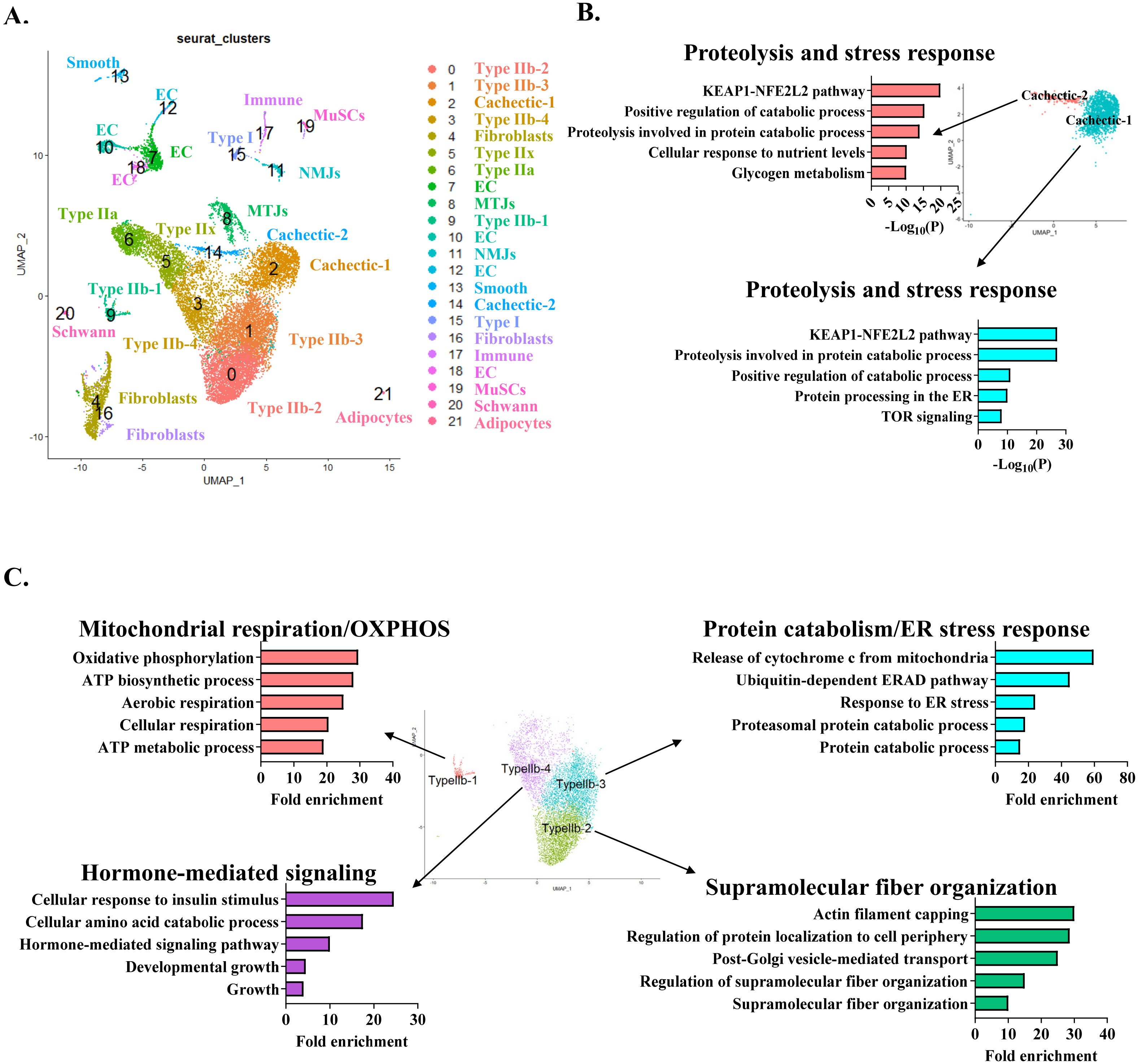
A.
seurat_clusters
UMAP_1
UMAP_2
0 Type IIb-2
1 Type IIb-3
2 Cachectic-1
3 Type IIb-4
4 Fibroblasts
5 Type IIx
6 Type IIa
7 EC
8 MTJs
9 Type IIb-1
10 EC
11 NMJs
12 EC
13 Smooth
14 Cachectic-2
15 Type I
16 Fibroblasts
17 Immune
18 EC
19 MuSCs
20 Schwann
21 Adipocytes
B.
Proteolysis and stress response
KEAP1-NFE2L2 pathway
Positive regulation of catabolic process
Proteolysis involved in protein catabolic process
Cellular response to nutrient levels
Glycogen metabolism
-Log10(P)
Cachectic-2
Cachectic-1
Proteolysis and stress response
KEAP1-NFE2L2 pathway
Proteolysis involved in protein catabolic process
Positive regulation of catabolic process
Protein processing in the ER
TOR signaling
-Log10(P)
C.
Mitochondrial respiration/OXPHOS
Oxidative phosphorylation
ATP biosynthetic process
Aerobic respiration
Cellular respiration
ATP metabolic process
Fold enrichment
TypeIIb-1
TypeIIb-4
TypeIIb-3
TypeIIb-2
Protein catabolism/ER stress response
Release of cytochrome c from mitochondria
Ubiquitin-dependent ERAD pathway
Response to ER stress
Proteasomal protein catabolic process
Protein catabolic process
Fold enrichment
Hormone-mediated signaling
Cellular response to insulin stimulus
Cellular amino acid catabolic process
Hormone-mediated signaling pathway
Developmental growth
Growth
Fold enrichment
Supramolecular fiber organization
Actin filament capping
Regulation of protein localization to cell periphery
Post-Golgi vesicle-mediated transport
Regulation of supramolecular fiber organization
Supramolecular fiber organization
Fold enrichment

Figure S1. Annotation of nuclear clusters to different cell types in skeletal muscle. (A) UMAP visualization of the integrated single-nucleus RNA-seq dataset from skeletal muscle of control and KPC tumor-bearing mice following unsupervised clustering. A total of 21 transcriptionally distinct clusters were resolved, each point representing a single nucleus, with colors indicating cluster identity. **(B)** Enrichment analysis of the two cachectic clusters to annotate their differences. **(C)** Enrichment analysis of the four type-IIb clusters to annotate their differences.

FIGURE S2

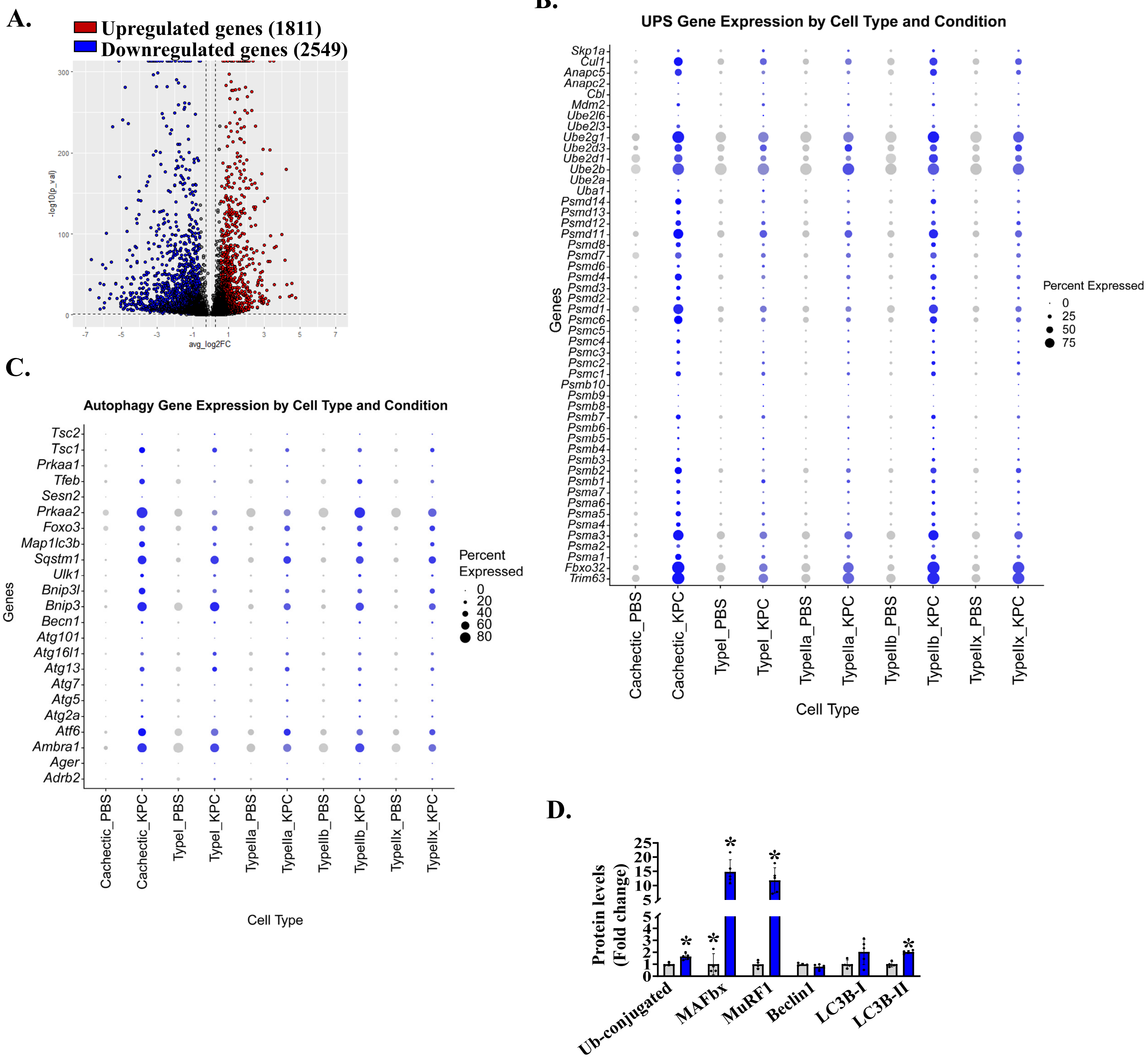

A.
Upregulated genes (1811)
Downregulated genes (2549)
-log10(p_val)
300
200
100
0
-7
-5
-3
-1
1
3
5
7
avg_log2FC
B.
UPS Gene Expression by Cell Type and Condition
Skp1a
Cul1
Anapc5
Anapc2
Cbl
Mdm2
Ube2l6
Ube2l3
Ube2g1
Ube2d3
Ube2d1
Ube2b
Ube2a
Uba1
Psmd14
Psmd13
Psmd12
Psmd11
Psmd8
Psmd7
Psmd6
Psmd4
Psmd3
Psmd2
Psmd1
Psmc6
Psmc5
Psmc4
Psmc3
Psmc2
Psmc1
Psmb10
Psmb9
Psmb8
Psmb7
Psmb6
Psmb5
Psmb4
Psmb3
Psmb2
Psmb1
Psma7
Psma6
Psma5
Psma4
Psma3
Psma2
Psma1
Fbxo32
Trim63
Genes
Percent Expressed
0
25
50
75
Cachectic_PBS
Cachectic_KPC
TypeI_PBS
TypeI_KPC
TypeIIa_PBS
TypeIIa_KPC
TypeIIb_PBS
TypeIIb_KPC
TypeIIx_PBS
TypeIIx_KPC
Cell Type
C.
Autophagy Gene Expression by Cell Type and Condition
Tsc2
Tsc1
Prkaa1
Tfeb
Sesn2
Prkaa2
Foxo3
Map1lc3b
Sqstm1
Ulk1
Bnip3l
Bnip3
Becn1
Atg101
Atg16l1
Atg13
Atg7
Atg5
Atg2a
Atf6
Ambra1
Ager
Adrb2
Genes
Percent Expressed
0
20
40
60
80
Cachectic_PBS
Cachectic_KPC
TypeI_PBS
TypeI_KPC
TypeIIa_PBS
TypeIIa_KPC
TypeIIb_PBS
TypeIIb_KPC
TypeIIx_PBS
TypeIIx_KPC
Cell Type
D.
Protein levels (Fold change)
25
20
15
10
5
5
4
3
2
1
0
Ub-conjugated
MAFbx
MuRF1
Beclin1
LC3B-I
LC3B-II

Figure S2. **Increased expression of UPS and autophagy-related genes in cachectic myonuclear clusters. (A)** Volcano plot showing differentially expressed genes in cachectic myonuclei compared with all control muscle nuclei, with 1,811 genes upregulated (red) and 2,549 genes downregulated (blue). **(B)** Dot plot showing the expression patterns of genes within the ubiquitin–proteasome system (UPS) gene set used for module scoring. **(C)** Dot plot showing the expression patterns of genes within the autophagy gene set used for module scoring. Dot size indicates the percentage of nuclei expressing each gene within a given fiber type, and dot color reflects average expression levels. **(D)** Quantification of the levels of total ubiquitinated proteins, MAFbx, MuRF1, Beclin1, LC3B-I/II.

FIGURE S3

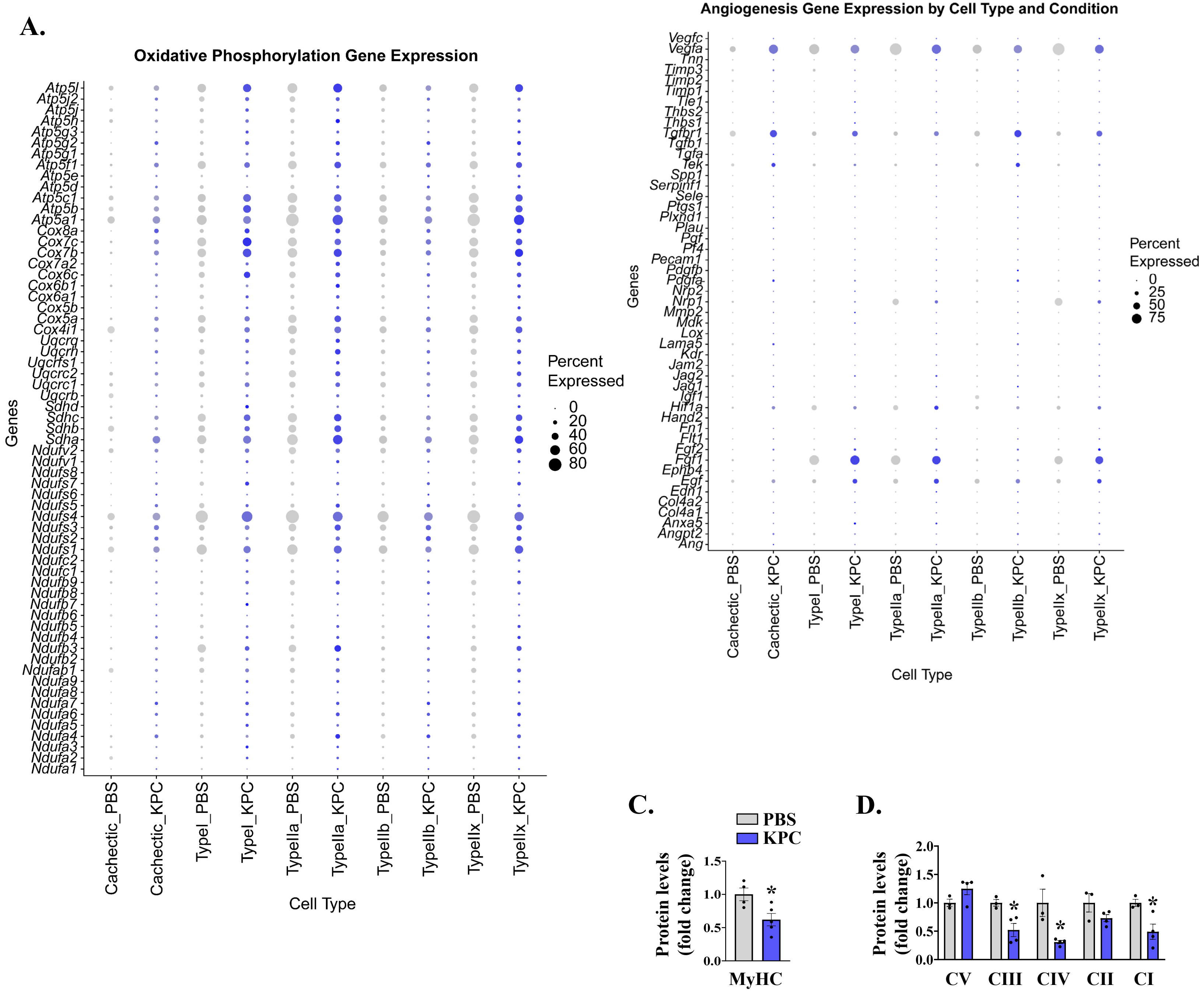
A.
Oxidative Phosphorylation Gene Expression
Genes
Atp5l, Atp5j2, Atp5j, Atp5h, Atp5g3, Atp5g2, Atp5g1, Atp5f1, Atp5e, Atp5d, Atp5c1, Atp5b, Atp5a1, Cox8a, Cox7c, Cox7b, Cox7a2, Cox6c, Cox6b1, Cox6a1, Cox5b, Cox5a, Cox4i1, Uqcrq, Uqcrh, Uqcrfs1, Uqcrc2, Uqcrc1, Uqcrb, Sdhd, Sdhc, Sdhb, Sdha, Ndufv2, Ndufv1, Ndufs8, Ndufs7, Ndufs6, Ndufs5, Ndufs4, Ndufs3, Ndufs2, Ndufs1, Ndufc2, Ndufc1, Ndufb9, Ndufb8, Ndufb7, Ndufb6, Ndufb5, Ndufb4, Ndufb3, Ndufb2, Ndufab1, Ndufa9, Ndufa8, Ndufa7, Ndufa6, Ndufa5, Ndufa4, Ndufa3, Ndufa2, Ndufa1
Percent Expressed
0
20
40
60
80
Cachectic_PBS, Cachectic_KPC, TypeI_PBS, TypeI_KPC, TypeIIa_PBS, TypeIIa_KPC, TypeIIb_PBS, TypeIIb_KPC, TypeIIx_PBS, TypeIIx_KPC
Cell Type
B.
Angiogenesis Gene Expression by Cell Type and Condition
Genes
Vegfc, Vegfa, Tnn, Timp3, Timp2, Timp1, Tie1, Thbs2, Thbs1, Tgfbr1, Tgfb1, Tgfa, Tek, Spp1, Serpinf1, Sele, Ptgs1, Plxnd1, Plau, Pgf, Pf4, Pecam1, Pdgfb, Pdgfa, Nrp2, Nrp1, Mmp2, Mdk, Lox, Lama5, Kdr, Jam2, Jag2, Jag1, Igf1, Hif1a, Hand2, Fn1, Flt1, Fgf2, Fgf1, Ephb4, Egf, Edn1, Col4a2, Col4a1, Anxa5, Angpt2, Ang
Percent Expressed
0
25
50
75
Cachectic_PBS, Cachectic_KPC, TypeI_PBS, TypeI_KPC, TypeIIa_PBS, TypeIIa_KPC, TypeIIb_PBS, TypeIIb_KPC, TypeIIx_PBS, TypeIIx_KPC
Cell Type
C.
PBS
KPC
Protein levels (fold change)
0.0
0.5
1.0
1.5
*
MyHC
D.
Protein levels (fold change)
0.0
0.5
1.0
1.5
2.0
*
*
*
CV
CIII
CIV
CII
CI

Figure S3. Repression in gene expression of molecules related to oxidative phosphorylation and angiogenesis. (A) Dot plot showing the expression patterns of genes within the oxidative phosphorylation gene set used for module scoring. **(B)** Dot plot showing the expression patterns of genes within the angiogenesis gene set used for module scoring. Dot size indicates the percentage of nuclei expressing each gene within a given fiber type, and dot color reflects average expression levels. Relative protein levels of **(C)** MyHC, and **(D)** mitochondrial oxidative phosphorylation (OXPHOS) complexes I-V.

FIGURE S4

A.

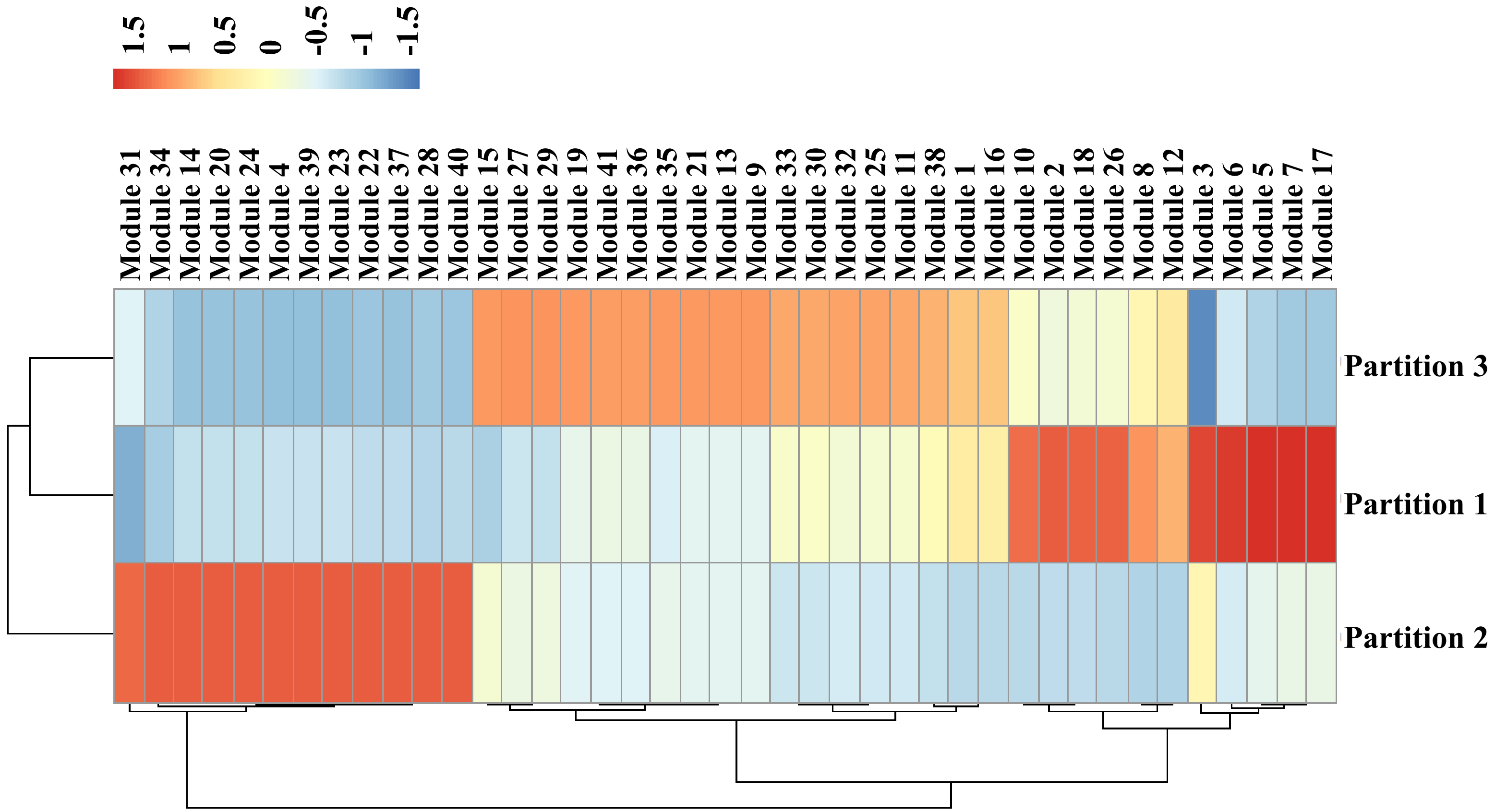
1.5
1
0.5
0
-0.5
-1
-1.5
Module 31
Module 34
Module 14
Module 20
Module 24
Module 4
Module 39
Module 23
Module 22
Module 37
Module 28
Module 40
Module 15
Module 27
Module 29
Module 19
Module 41
Module 36
Module 35
Module 21
Module 13
Module 9
Module 33
Module 30
Module 32
Module 25
Module 11
Module 38
Module 1
Module 16
Module 10
Module 2
Module 18
Module 26
Module 8
Module 12
Module 3
Module 6
Module 5
Module 7
Module 17
Partition 3
Partition 1
Partition 2

Figure S4. Gene co-expression modules of cachectic myonuclei. Heatmap showing 41 gene modules identified by Louvain community detection method grouped into 3 partitions in myonuclear clusters of KPC tumor-bearing mice.

Figure S5

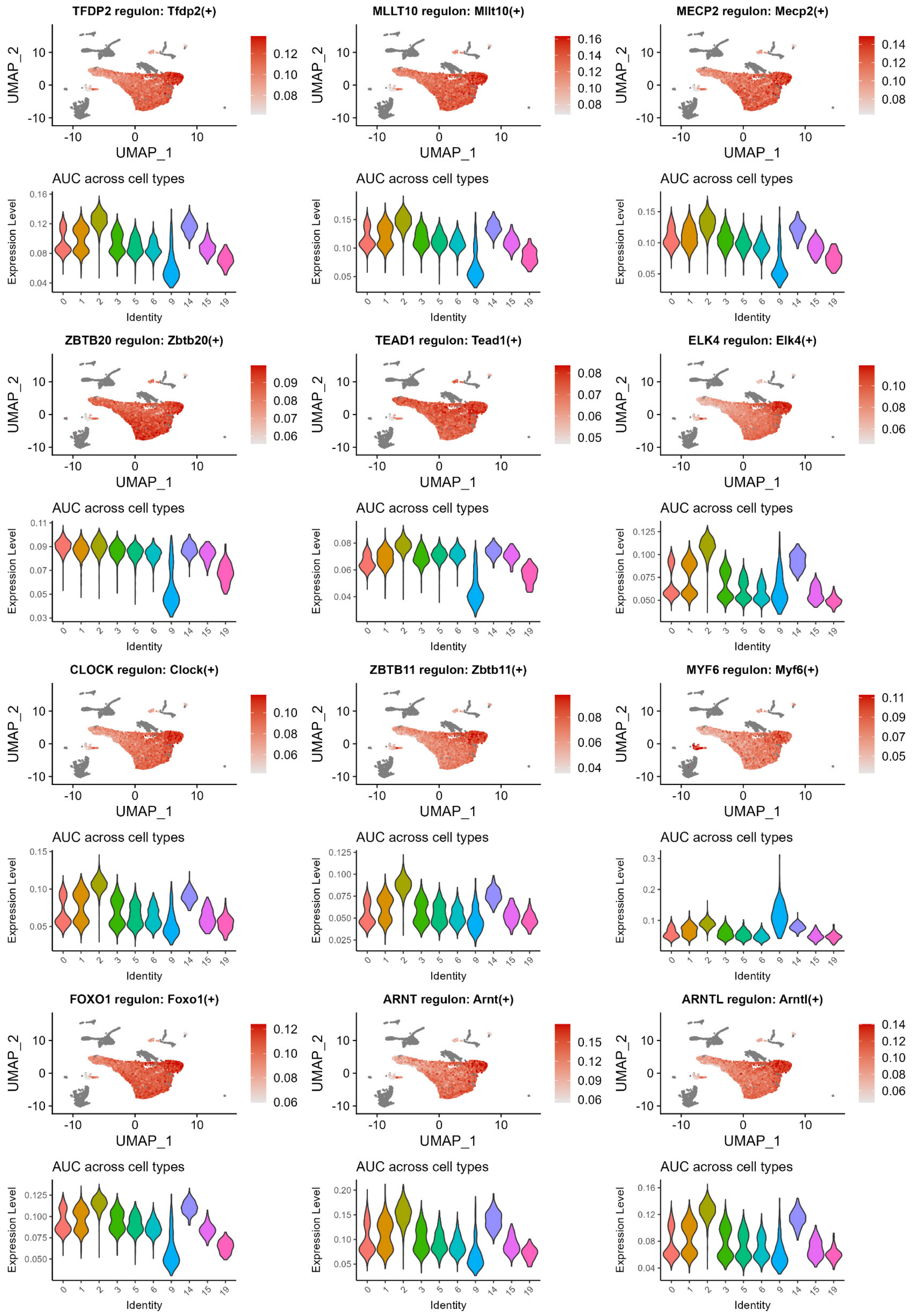

TFDP2 regulon: Tfdp2(+)
MLLT10 regulon: Mllt10(+)
MECP2 regulon: Mecp2(+)
ZBTB20 regulon: Zbtb20(+)
TEAD1 regulon: Tead1(+)
ELK4 regulon: Elk4(+)
CLOCK regulon: Clock(+)
ZBTB11 regulon: Zbtb11(+)
MYF6 regulon: Myf6(+)
FOXO1 regulon: Foxo1(+)
ARNT regulon: Arnt(+)
ARNTL regulon: Arntl(+)
UMAP_1
UMAP_2
AUC across cell types
Expression Level
Identity

Figure S5. Translation initiation and mTOR-related transcription factors activity in myonuclei. UMAP visualization of regulon activity for mTOR- and translation-associated transcription factors, with violin plots showing cluster-specific activation patterns.

Figure S6

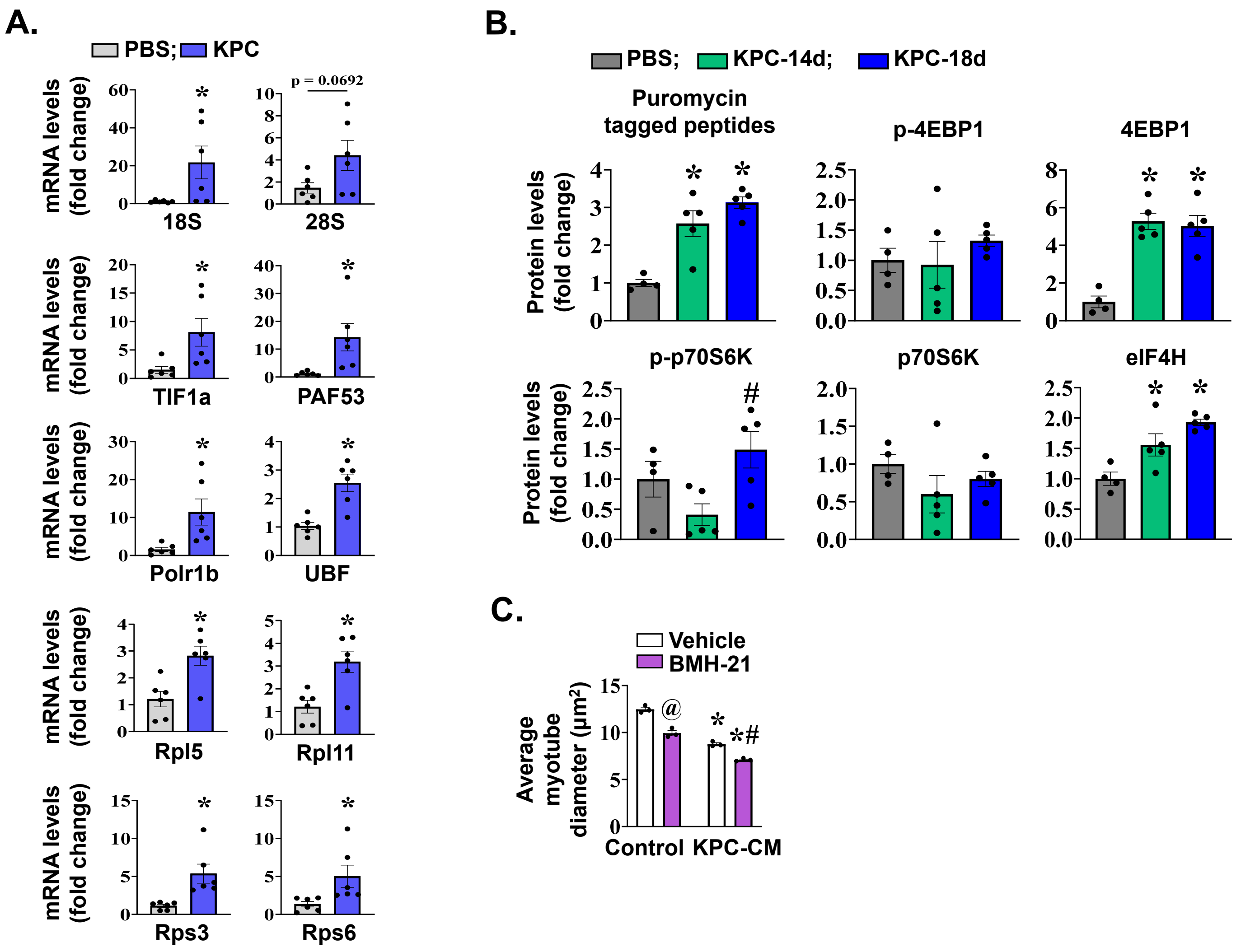
A.
PBS; KPC
mRNA levels (fold change)
p = 0.0692
18S
28S
TIF1a
PAF53
Polr1b
UBF
Rpl5
Rpl11
Rps3
Rps6
B.
PBS; KPC-14d; KPC-18d
Puromycin tagged peptides
p-4EBP1
4EBP1
Protein levels (fold change)
p-p70S6K
p70S6K
eIF4H
C.
Vehicle
BMH-21
Average myotube diameter (µm²)
Control
KPC-CM

Figure S6. KPC tumor growth induces alterations in markers of ribosome biogenesis and translation initiation. (A) Relative mRNA levels of ribosome biogenesis-related molecules in skeletal muscle of control and KPC tumor-bearing mice. n=4-6 per group. Data are presented as mean ± SEM. $^{*}p \leq 0.05$, vs. control mice (unpaired Student t test). **(B)** Quantification of levels of puromycin-tagged peptides and levels of p-4EBP1, 4EBP1, p-70S6K, p70S6K, eIF4H, and GAPDH in TA muscle of control and KPC tumor-bearing mice after 14 and 18 days of KPC cells injection in pancreas. **(C)** Average myotube diameter in cultures treated with vehicle alone or BMH-21 and incubated with or without KPC cells conditioned medium (KPC-CM). n=3 per group. Data are presented as mean ± SEM. $^{*}p \leq 0.05$, vs. corresponding control cultures, $^{@}p \leq 0.05$, vs. vehicle-treated control cultures, and $^{\#}p \leq 0.05$, vs. control cultures incubated in KPC-CM (two-way ANOVA and Tukey's multiple comparison test).

Figure S7

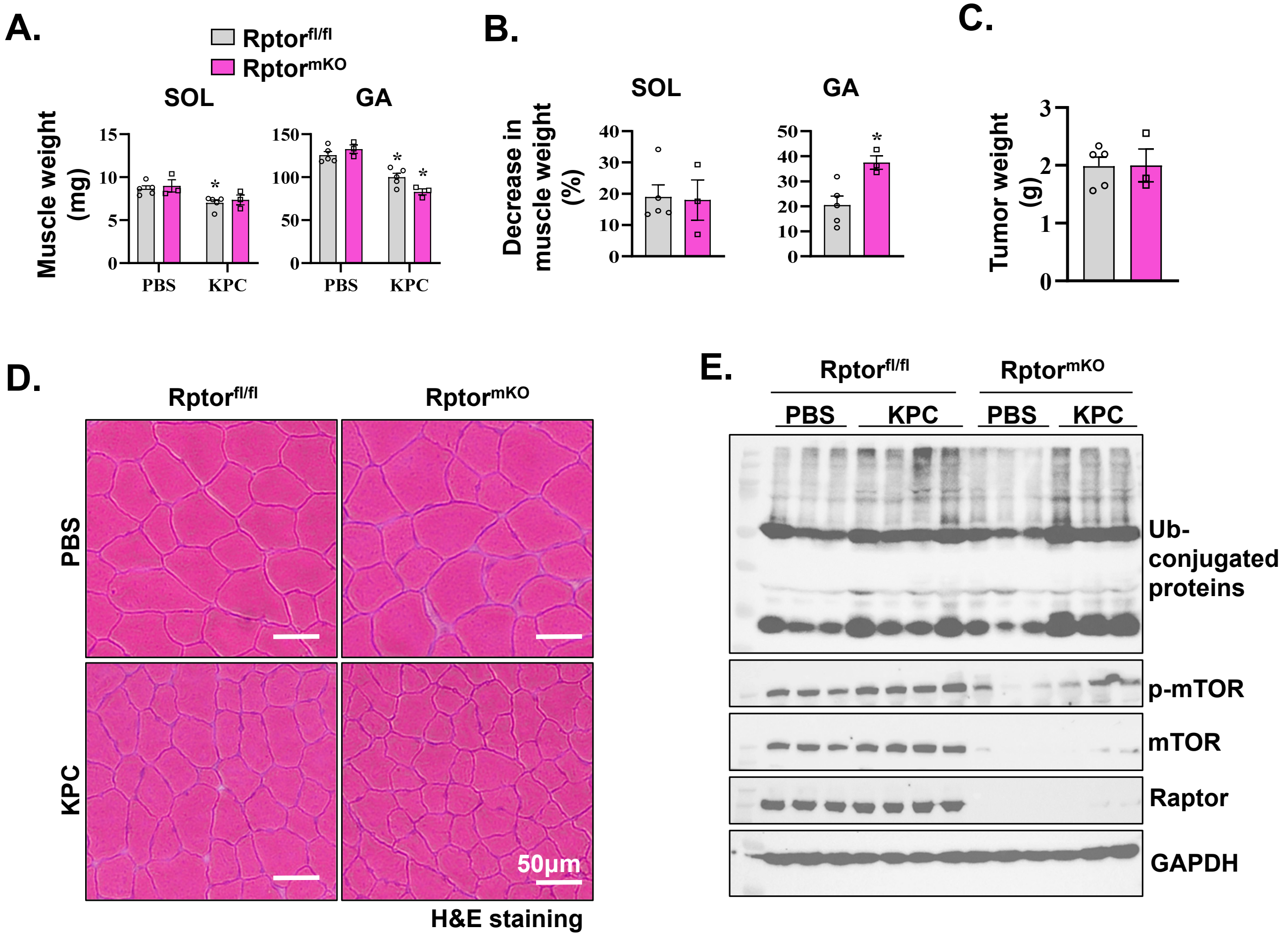
A.
Rptor^fl/fl
Rptor^mKO
SOL
GA
Muscle weight (mg)
PBS
KPC
B.
Decrease in muscle weight (%)
C.
Tumor weight (g)
D.
PBS
KPC
50μm
H&E staining
E.
Ub-conjugated proteins
p-mTOR
mTOR
Raptor
GAPDH

Figure S7. Targeted deletion of Raptor exacerbates muscle loss in response to KPC tumor growth. (A) Wet muscle weight of soleus (SOL), and gastrocnemius (GA) muscle of control and KPC tumor-bearing $Rptor^{fl/fl}$ and $Rptor^{mko}$ mice. **(B)** Tumor-induced decrease in SOL and GA muscle weight in $Rptor^{fl/fl}$ and $Rptor^{mKO}$ mice. n=3-5 per group. Data are presented as mean ± SEM. *$p \leq 0.05$, vs. corresponding control mice (unpaired Student *t* test or two-way ANOVA and Tukey's multiple comparison test). **(C)** Wet weight of tumor in $Rptor^{fl/fl}$ and $Rptor^{mKO}$ mice. **(D)** Representative images of TA muscle cross-sections after H&E staining. Scale bar, 50 µm. **(E)** Immunoblots showing levels of indicated proteins in skeletal muscle of control and KPC tumor-bearing $Rptor^{fl/fl}$ and $Rptor^{mKO}$ mice.

Figure S8

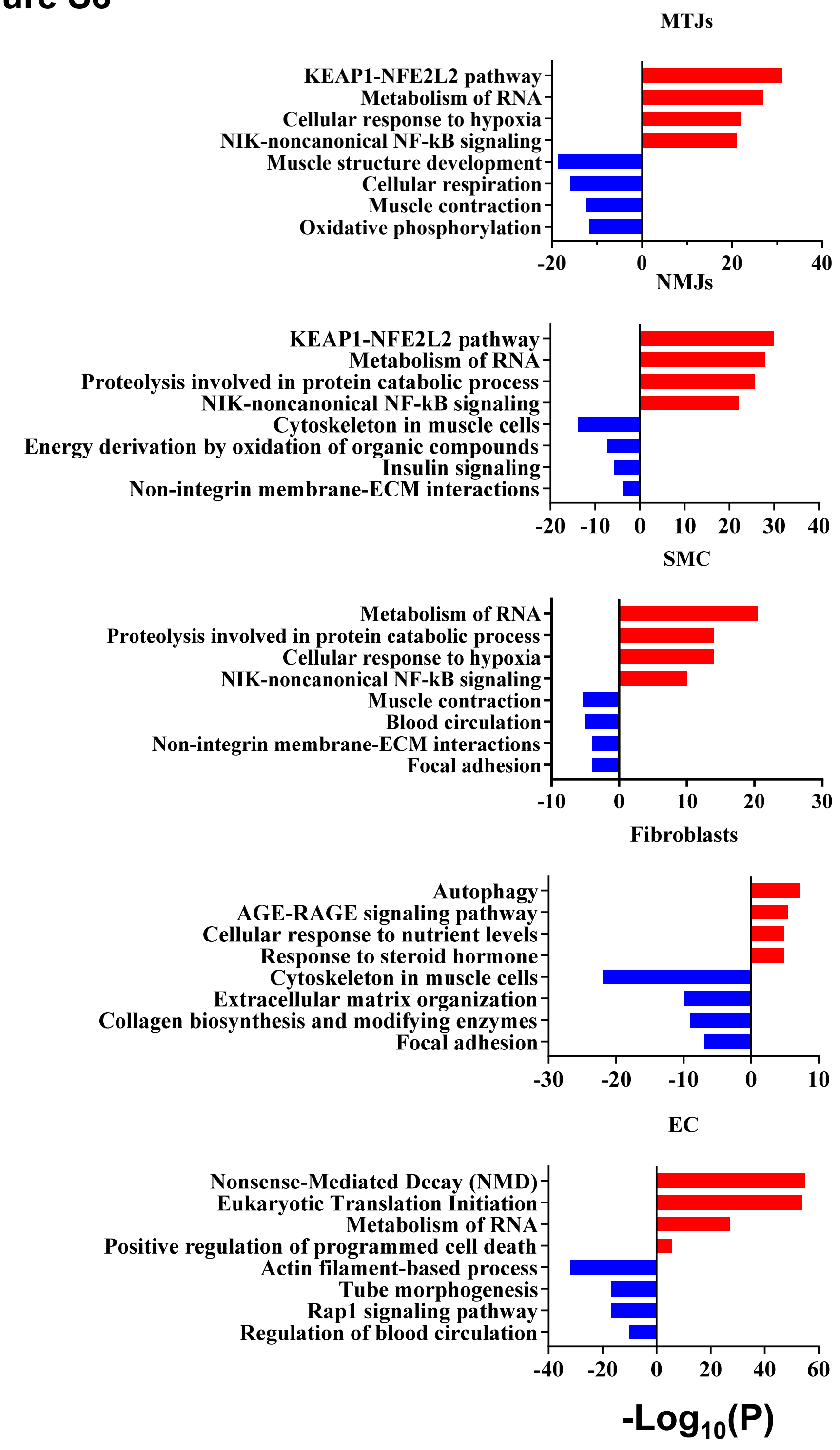

MTJs
KEAP1-NFE2L2 pathway
Metabolism of RNA
Cellular response to hypoxia
NIK-noncanonical NF-kB signaling
Muscle structure development
Cellular respiration
Muscle contraction
Oxidative phosphorylation
-20
0
20
40
NMJs
KEAP1-NFE2L2 pathway
Metabolism of RNA
Proteolysis involved in protein catabolic process
NIK-noncanonical NF-kB signaling
Cytoskeleton in muscle cells
Energy derivation by oxidation of organic compounds
Insulin signaling
Non-integrin membrane-ECM interactions
-20
-10
0
10
20
30
40
SMC
Metabolism of RNA
Proteolysis involved in protein catabolic process
Cellular response to hypoxia
NIK-noncanonical NF-kB signaling
Muscle contraction
Blood circulation
Non-integrin membrane-ECM interactions
Focal adhesion
-10
0
10
20
30
Fibroblasts
Autophagy
AGE-RAGE signaling pathway
Cellular response to nutrient levels
Response to steroid hormone
Cytoskeleton in muscle cells
Extracellular matrix organization
Collagen biosynthesis and modifying enzymes
Focal adhesion
-30
-20
-10
0
10
EC
Nonsense-Mediated Decay (NMD)
Eukaryotic Translation Initiation
Metabolism of RNA
Positive regulation of programmed cell death
Actin filament-based process
Tube morphogenesis
Rap1 signaling pathway
Regulation of blood circulation
-40
-20
0
20
40
60
$-Log_{10}(P)$

Figure S8. **Pathway analysis of differentially expressed genes in non-myogenic nuclear clusters**. Bar diagrams presented here demonstrate up-regulated and downregulated pathways in the nuclei of MTJs, NMJs. SMC, fibroblasts, and endothelial (EC) cells.

Figure S9

A.

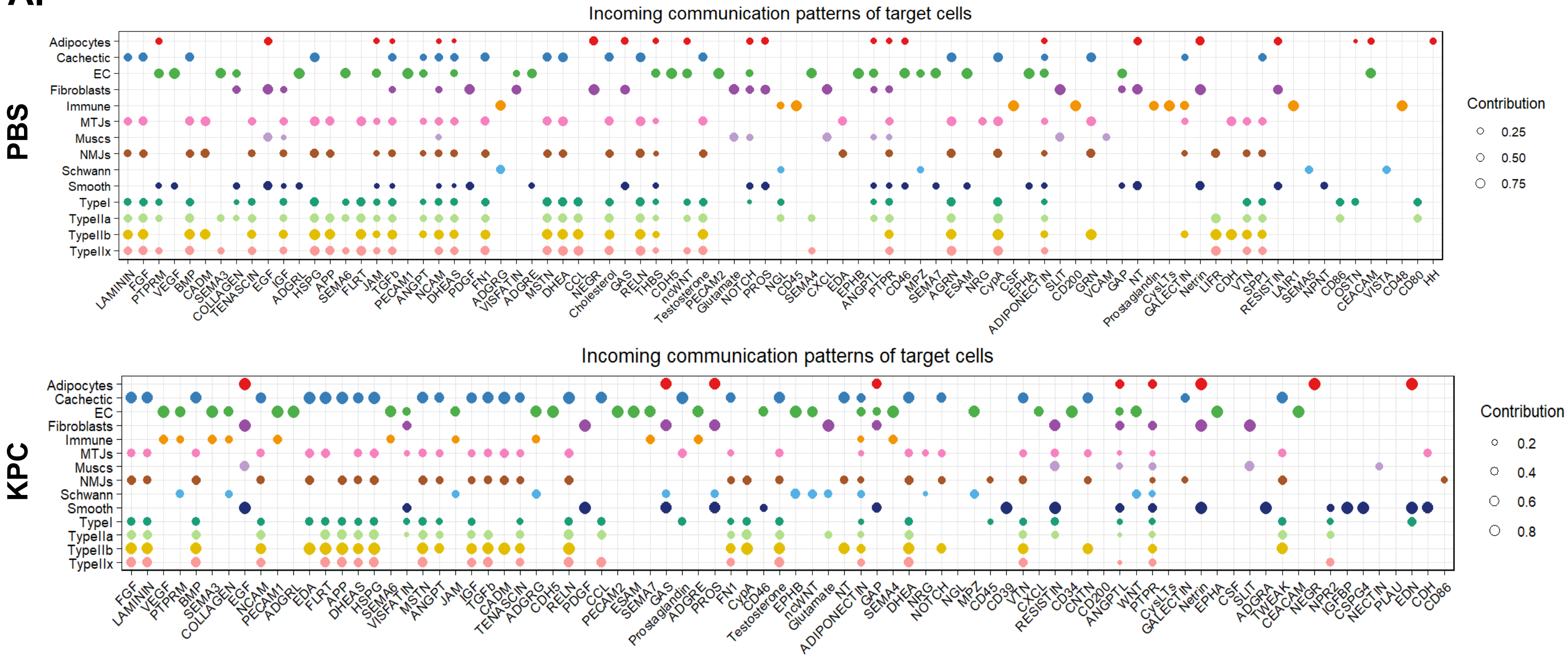
Incoming communication patterns of target cells
PBS
KPC
Contribution
0.25
0.50
0.75
0.2
0.4
0.6
0.8
Adipocytes
Cachectic
EC
Fibroblasts
Immune
MTJs
Muscs
NMJs
Schwann
Smooth
TypeI
TypeIIa
TypeIIb
TypeIIx

B.

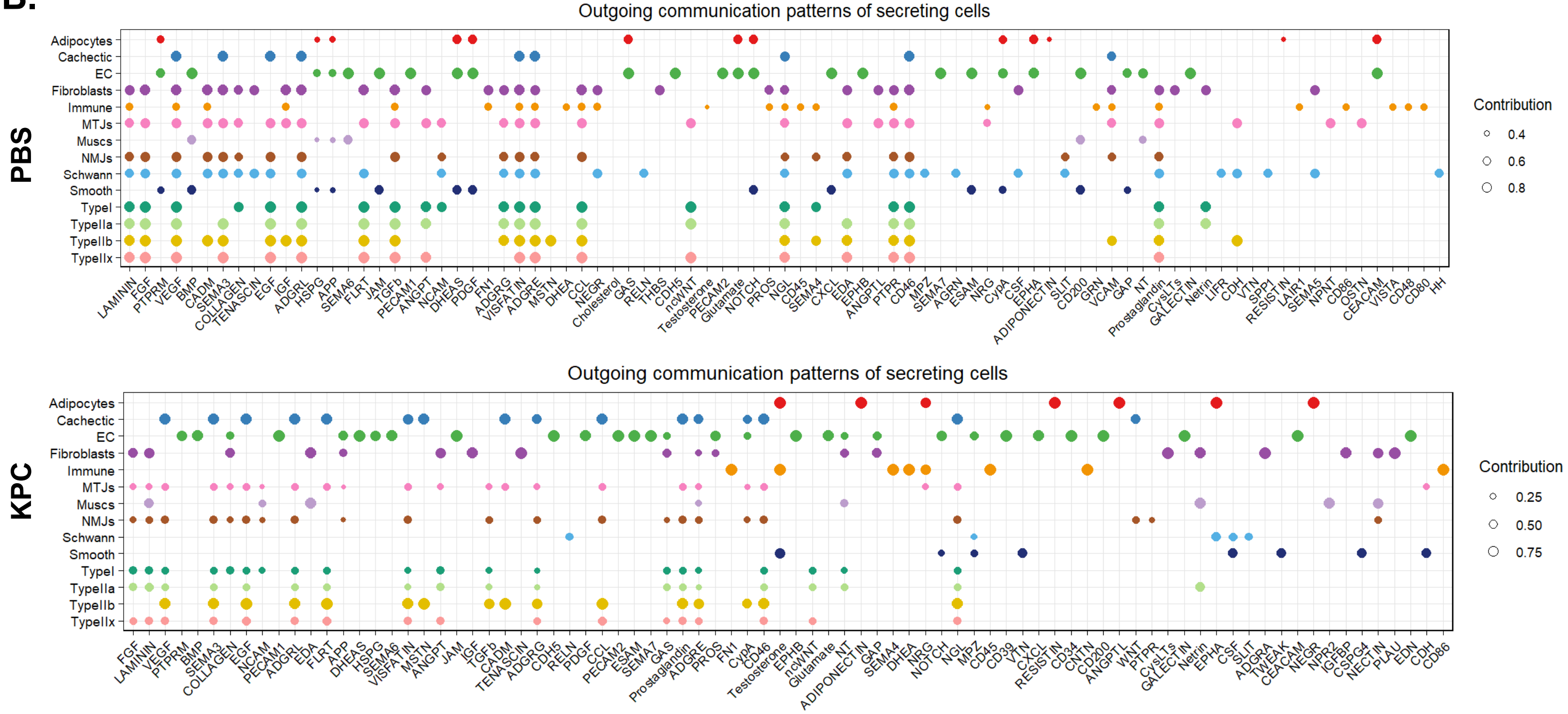
Outgoing communication patterns of secreting cells
PBS
KPC
Contribution
0.4
0.6
0.8
0.25
0.50
0.75
Adipocytes
Cachectic
EC
Fibroblasts
Immune
MTJs
Muscs
NMJs
Schwann
Smooth
TypeI
TypeIIa
TypeIIb
TypeIIx

Figure S9. Changes in intercellular communications within skeletal muscle of tumor-bearing mice. (A) Dot plots showing incoming signaling patterns across all cell types in PBS control and KPC tumor-bearing muscle. **(B)** Dot plots showing outgoing signaling patterns across all cell types in PBS control and KPC tumor-bearing muscle.

Figure S10

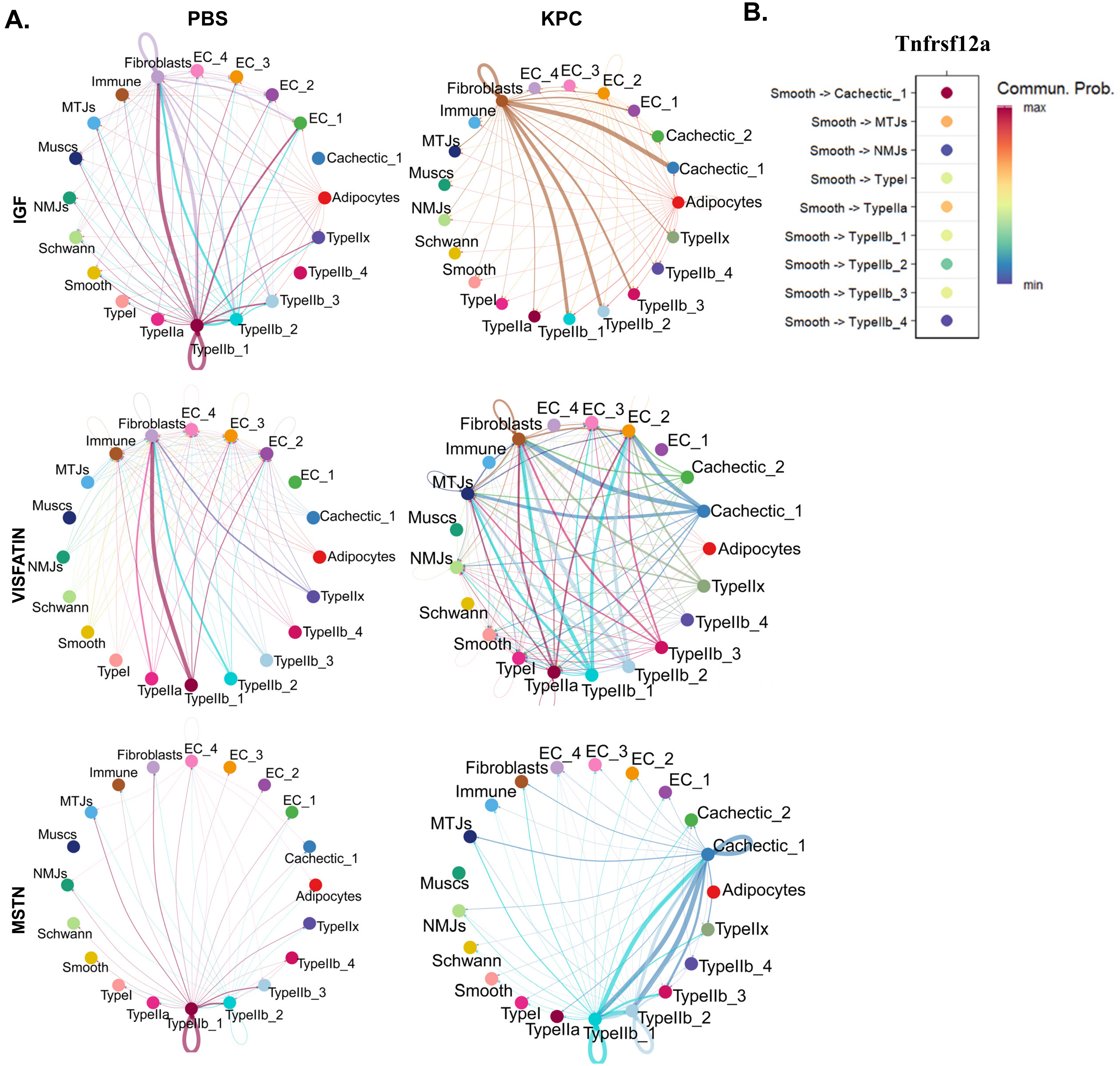

A.
PBS
KPC
IGF
VISFATIN
MSTN
Fibroblasts
EC_4
EC_3
EC_2
EC_1
Immune
MTJs
Muscs
NMJs
Schwann
Smooth
TypeI
TypeIIa
TypeIIb_1
TypeIIb_2
TypeIIb_3
TypeIIb_4
TypeIIx
Adipocytes
Cachectic_1
Cachectic_2
B.
Tnfrsf12a
Smooth -> Cachectic_1
Smooth -> MTJs
Smooth -> NMJs
Smooth -> TypeI
Smooth -> TypeIIa
Smooth -> TypeIIb_1
Smooth -> TypeIIb_2
Smooth -> TypeIIb_3
Smooth -> TypeIIb_4
Commun. Prob.
max
min

Figure S10. Intercellular signaling changes in cachectic muscle. (A) Circle plot illustrating intercellular communication mediated by IGF, MSTN, VISFATIN in control (PBS) and KPC tumor-bearing mice. **(B)** Dot plot showing interaction of TWEAK with its receptor Tnfrsf12a in different myonuclei of KPC tumor-bearing mice identified by CellChat analysis of snRNA-Seq dataset.

Figure S11

A.

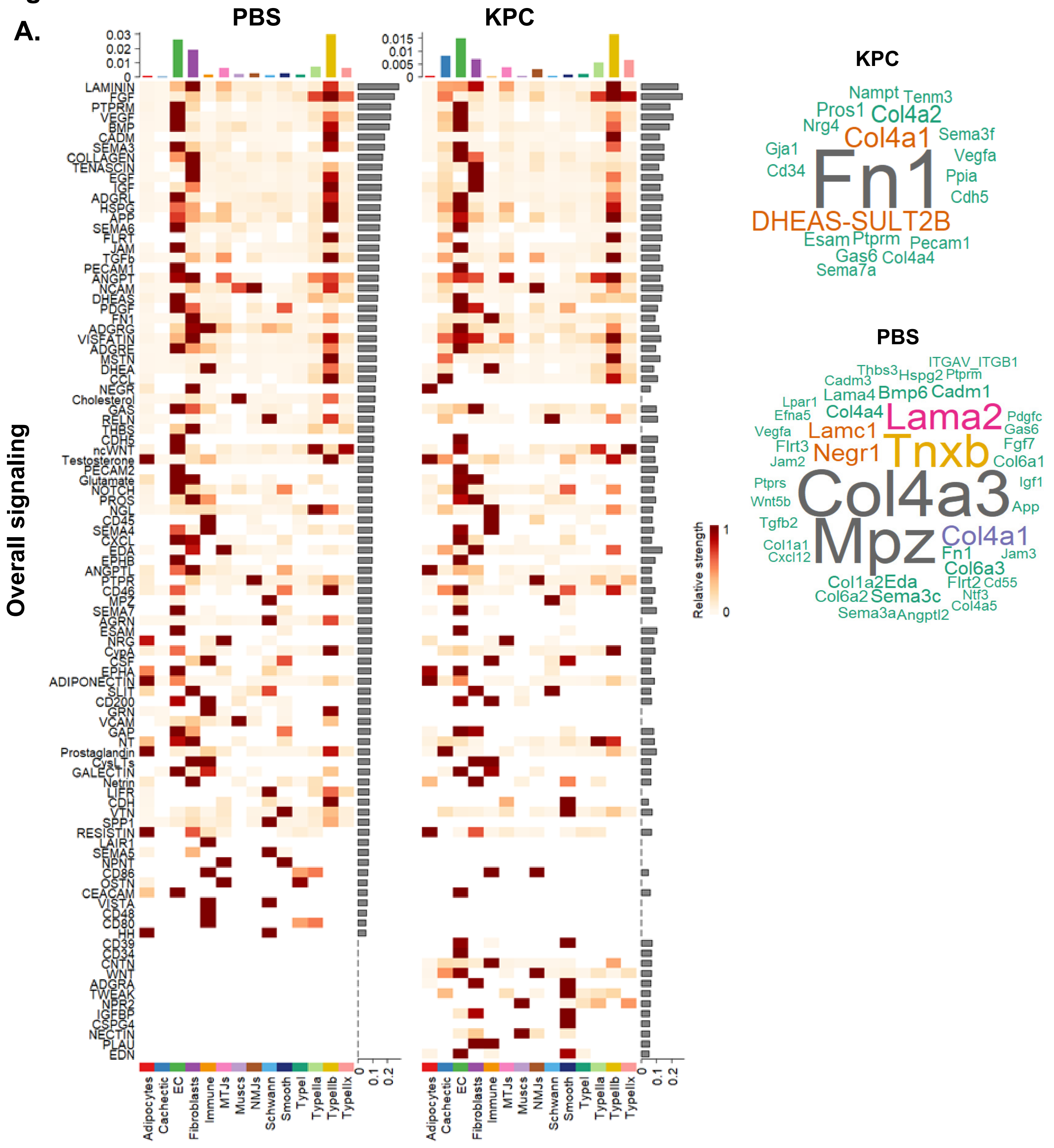
PBS
KPC
Overall signaling
Relative strength
1
0
0.03
0.02
0.01
0.015
0.005
0.1
0.2
LAMININ
FGF
PTPRM
VEGF
BMP
CADM
SEMA3
COLLAGEN
TENASCIN
EGF
IGF
ADGRL
HSPG
APP
SEMA6
FLRT
JAM
TGFb
PECAM1
ANGPT
NCAM
DHEAS
PDGF
FN1
ADGRG
VISFATIN
ADGRE
MSTN
DHEA
CCL
NEGR
Cholesterol
GAS
RELN
THBS
CDH5
ncWNT
Testosterone
PECAM2
Glutamate
NOTCH
PROS
NGL
CD45
SEMA4
CXCL
EDA
EPHB
ANGPTL
PTPR
CD46
MPZ
SEMA7
AGRN
ESAM
NRG
CypA
CSF
EPHA
ADIPONECTIN
SLIT
CD200
GRN
VCAM
GAP
NT
Prostaglandin
CysLTs
GALECTIN
Netrin
LIFR
CDH
VTN
SPP1
RESISTIN
LAIR1
SEMA5
NPNT
CD86
OSTN
CEACAM
VISTA
CD48
CD80
HH
CD39
CD34
CNTN
WNT
ADGRA
TWEAK
NPR2
IGFBP
CSPG4
NECTIN
PLAU
EDN
Adipocytes
Cachectic
EC
Fibroblasts
Immune
MTJs
Muscs
NMJs
Schwann
Smooth
TypeI
TypeIIa
TypeIIb
TypeIIx
KPC
Nampt
Tenm3
Pros1
Col4a2
Nrg4
Col4a1
Sema3f
Gja1
Vegfa
Fn1
Ppia
Cd34
Cdh5
DHEAS-SULT2B
Esam
Ptprm
Pecam1
Gas6
Col4a4
Sema7a
PBS
Thbs3
ITGAV_ITGB1
Cadm3
Hspg2
Ptprm
Lpar1
Lama4
Bmp6
Cadm1
Efna5
Col4a4
Lama2
Pdgfc
Vegfa
Lamc1
Gas6
Flrt3
Negr1
Tnxb
Fgf7
Jam2
Col6a1
Ptprs
Col4a3
Igf1
Wnt5b
App
Tgfb2
Mpz
Col4a1
Col1a1
Fn1
Jam3
Cxcl12
Col6a3
Col1a2
Eda
Flrt2
Cd55
Col6a2
Sema3c
Ntf3
Col4a5
Sema3a
Angptl2

Figure S11. Alterations in inferred signaling flow in skeletal muscle of KPC tumor-bearing mice. (A) Heatmaps of overall signaling (population-weighted) in nuclear clusters of control and KPC tumor-bearing mice. **(B)** Word cloud visualization of ligand/receptor activity highlights dominant receptor or ligand in control and KPC tumor-bearing mice.

Figure S12

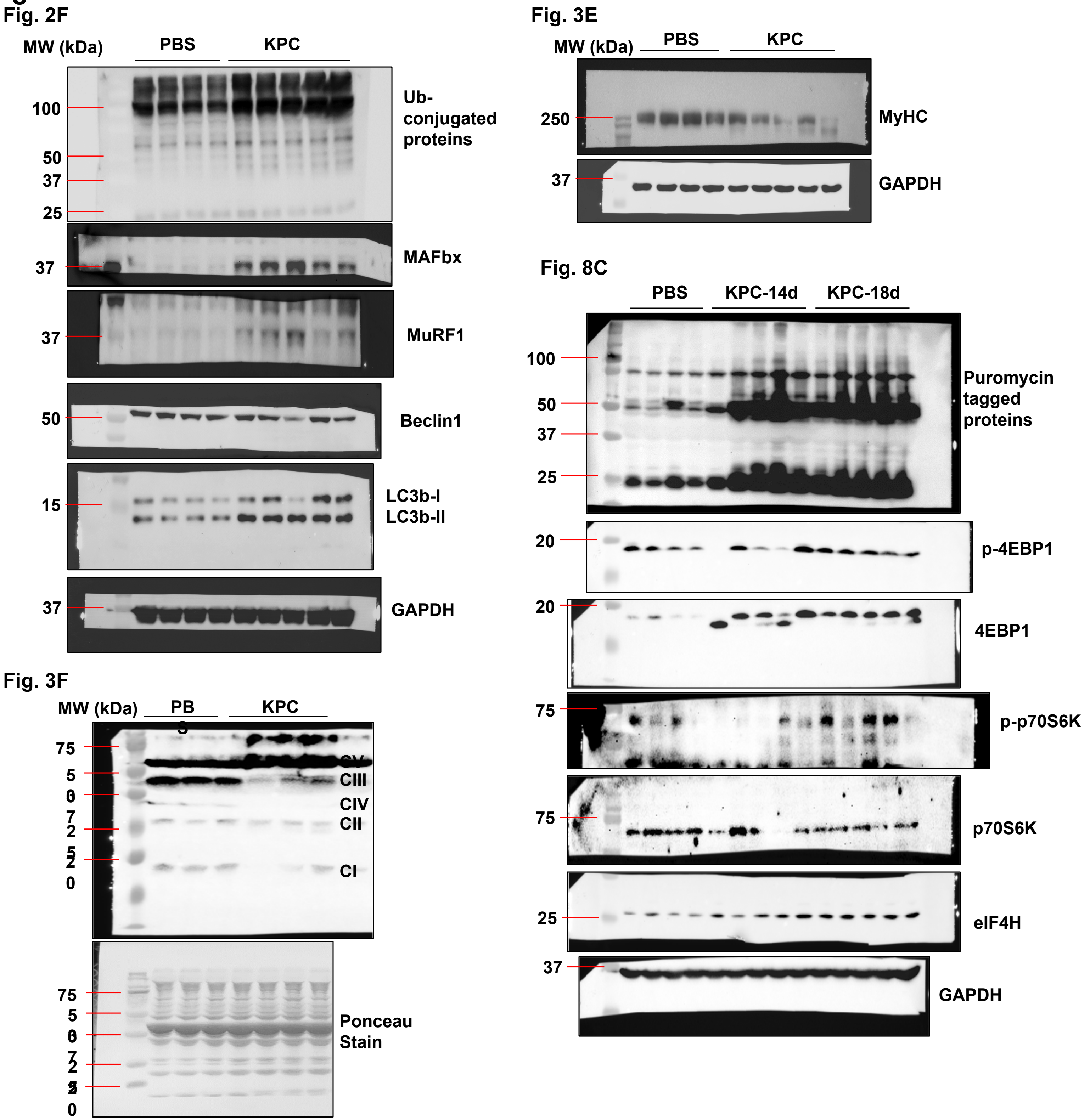

Fig. 2F
MW (kDa)
PBS
KPC
100
50
37
25
Ub-conjugated proteins
37
MAFbx
37
MuRF1
50
Beclin1
15
LC3b-I
LC3b-II
37
GAPDH
Fig. 3F
MW (kDa)
PBS
KPC
75
CV
CIII
CIV
CII
CI
75
Ponceau Stain
Fig. 3E
MW (kDa)
PBS
KPC
250
MyHC
37
GAPDH
Fig. 8C
PBS
KPC-14d
KPC-18d
100
50
37
25
Puromycin tagged proteins
20
p-4EBP1
20
4EBP1
75
p-p70S6K
75
p70S6K
25
eIF4H
37
GAPDH

Figure S12 (continuation)

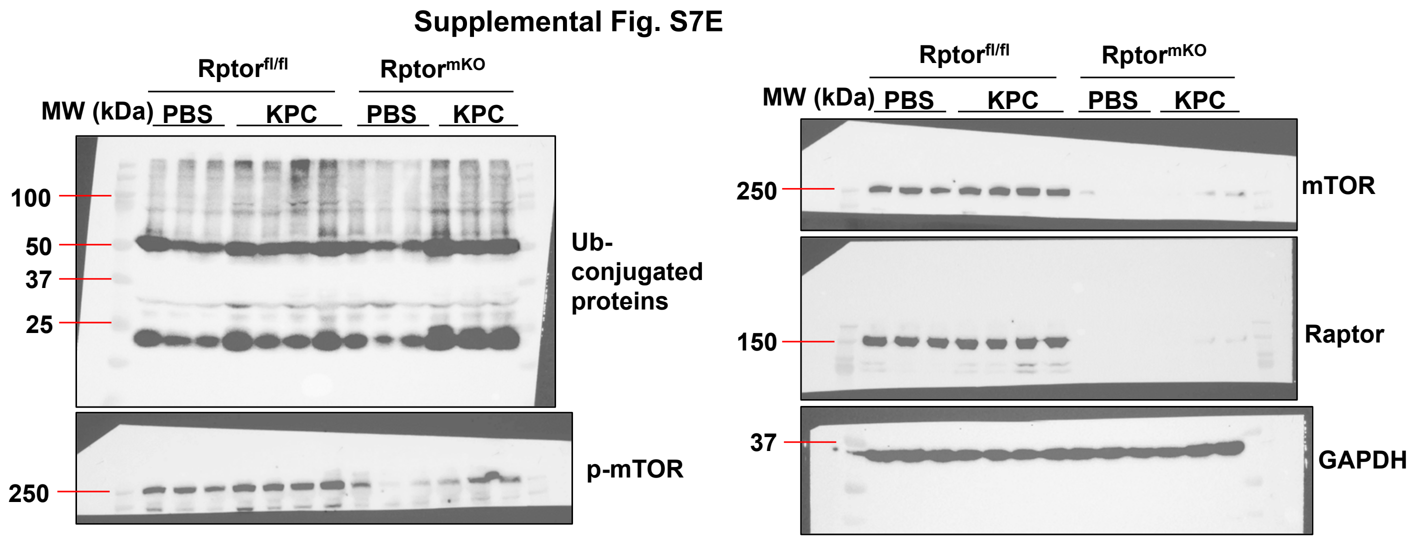

Figure S12. Uncropped western blot gel images. Original western blot images of immunoblots presented in the main and supplementary figures.

Table S1. List of antibodies used for Western blot (WB) and Immunofluorescence (IF).

Antibody	Source and Catalog no.	Analysis
Anti-MAFbx	ECM Biosciences #AP2041	WB
Anti-MuRF1	R&D Systems #AF5366	WB
Anti-Beclin1	Cell Signaling #3495S	WB
Anti-LC3b	Cell Signaling #2775S	WB
Anti-GAPDH	Cell Signaling #5174S	WB
Anti-Type I MyHC	DSHB #BA-D5	IF
Anti-Type IIa MyHC	DSHB #SC-71	IF
Anti-Type IIb MyHC	DSHB #BF-F3	IF
Anti-mTOR	Cell Signaling #2972	WB
Anti-p-mTOR	Cell Signaling #2971S	WB
Anti-Raptor	Cell Signaling #2280	WB
Anti-Puromycin	Millipore Sigma #MABE343	WB
Anti-4EBP1	Cell Signaling #9452S	WB
Anti-p-4EBP1	Cell Signaling #2972	WB
Anti-p70S6K	Cell Signaling #9202S	WB
Anti-p-p70S6K	Cell Signaling #9208S	WB
Anti-eIF4H	Cell Signaling #3469T	WB
Anti-Laminin	Sigma #L9393	IF
Anti-rabbit IgG	Cell Signaling #7074S	WB
Anti-mouse IgG	Cell Signaling #7076S	WB
Anti-goat IgG	Invitrogen #A15999	WB
Anti-mouse IgG1 AF568	Invitrogen #A21124	IF
Anti-rabbit IgG AF488	Invitrogen #A11034	IF
Goat anti-Mouse IgG2b AF350	Life Technologies #A21140	IF
Goat anti-Mouse IgG1 AF568	Life Technologies #A21124	IF
Goat anti-Mouse IgM AF488	Life Technologies #A21042	IF

Table S2. List of primers used for PCR/qRT-PCR analysis

Name	Forward primer (5′-3′)	Reverse primer (5′-3′)
β-actin	CAGGCATTGCTGACAGGATG	TGCTGATCCACATCTGCTGG
18s	CGGCTACCACATCCAAGGAA	GCTGGAATTACCGCGGCT
28s	TCATCAGACCCCAGAAAAGG	GATTCGGCAGGTGAGTTGTT
TIF1a	ATTCCCGTTTGTGAGGAAGTCCGA	TATCCTGCCGCGATACACTCACAT
PAF53	TCAGAACAAGACTTTCAGGGACAA	CTGCTTGGTGCTTCCAAAGG
Polr1b	TGGGAATCTGCGTTCTAAAAC	TTCAGCTTGTCAGCCACAACA
UBF	CGCGCAGCATACAAAGAATAC	GTTTGGGCCTCGGAGCTT
Rpl5	GGAGGTGAATGGAGGTGAATA	AGTTGTAGTTCGGGCAAGAC
Rpl11	TGCCTCAATATCTGCGTCGG	TTCTCCGGATGCCAAAGGAC
Rps3	CAGAGAAAGTGGCCACAAGA	TGAACCGAAGCACACCATAG
Rps6	CATGGGGGAGTTGGACCATA	TGGCCCTCTGTTCACACTA
Arrdc3	GCATAAAGGGCATTTGGTGGT	ATGGTAGTGAGTGCCCAAGG
Sik1	CCAAACACCTCTGGAAGGAA	CCTTTCCTCTCCCTTAATGACC
Sesn1	CTATTTGGCTGAGTGCTGCTA	GTCTTGACTGAGCTGTGAGTAA
Retreg1	CCATGAGTGATGTGGTCATTGG	AGACTGCAGTGGCAGAGTAT
Egln3	GAATCCCACAAGCAGCATAGA	GACAGTTCTTCCTGGACACTTT
Acss1	GCTGTGATCTGGAAGGAGTTT	ATGGGACCAGAGGCTTAGTA
Rgcc	CGAAGACTTCATTGCCGATCT	TCTCCCACAGCAGATTCTAGT
Irs2	TTGCTGTGTAGCTGAATCCC	ATATGCCATCTGTGTCCTGTG

Supplemental References

1. Hindi L, McMillan JD, Afroze D, Hindi SM, and Kumar A. Isolation, Culturing, and Differentiation of Primary Myoblasts from Skeletal Muscle of Adult Mice. *Bio Protoc.* 2017;7(9):e2248-e.

2. Roy A, and Kumar A. Supraphysiological activation of TAK1 promotes skeletal muscle growth and mitigates neurogenic atrophy. *Nat Commun.* 2022;13(1):2201.

3. Hindi SM, Sato S, Xiong G, Bohnert KR, Gibb AA, Gallot YS, et al. TAK1 regulates skeletal muscle mass and mitochondrial function. *JCI Insight.* 2018;3(3).

4. Jin S, Plikus MV, and Nie Q. CellChat for systematic analysis of cell-cell communication from single-cell transcriptomics. *Nat Protoc.* 2025;20(1):180-219.

5. Aibar S, Gonzalez-Blas CB, Moerman T, Huynh-Thu VA, Imrichova H, Hulselmans G, et al. SCENIC: single-cell regulatory network inference and clustering. *Nat Methods.* 2017;14(11):1083-6.

6. Hao Y, Stuart T, Kowalski MH, Choudhary S, Hoffman P, Hartman A, et al. Dictionary learning for integrative, multimodal and scalable single-cell analysis. *Nat Biotechnol.* 2024;42(2):293-304.
