# Supplementary material for: Fiber-type vulnerability and proteostasis reprogramming in skeletal muscle during pancreatic cancer cachexia
Source: JCI Insight. 2026 Jan 27;11(6):e200396. doi: 10.1172/jci.insight.200396 (PMC13043100; doi:10.1172/jci.insight.200396)

Unedited Blot and Gel Images

Fig. 2F

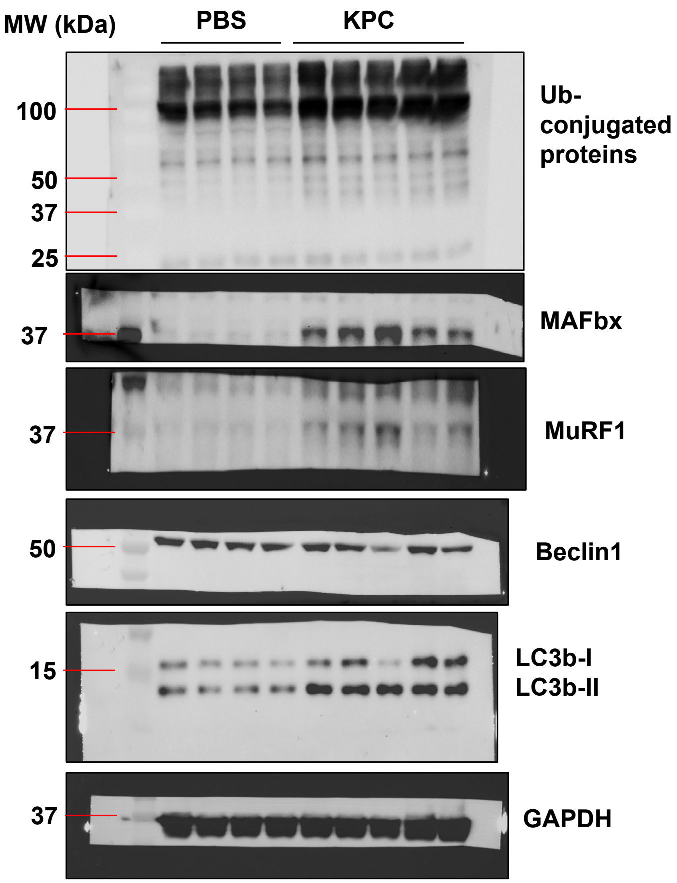

Fig. 3E

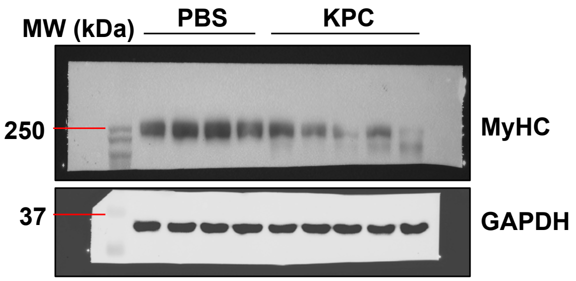

Fig. 8C

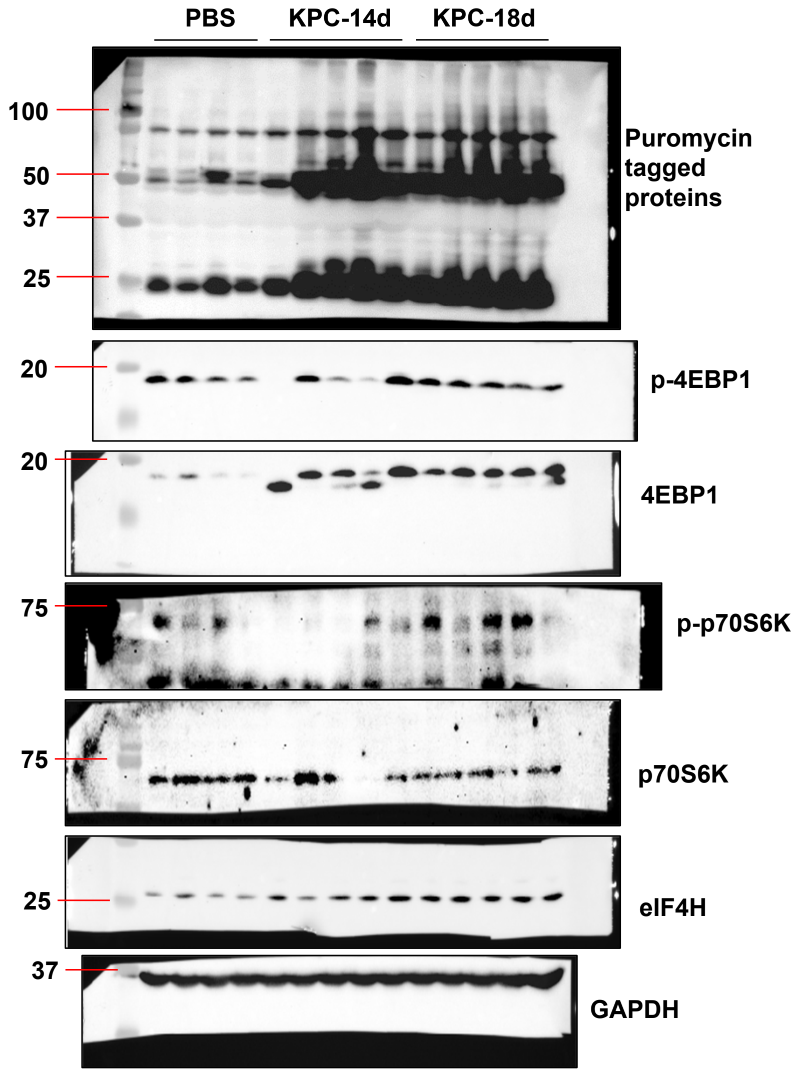

Fig. 3F

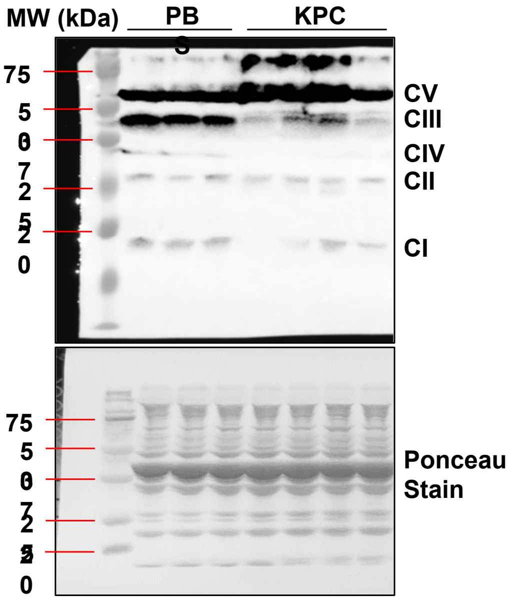

Supplemental Fig. S7E

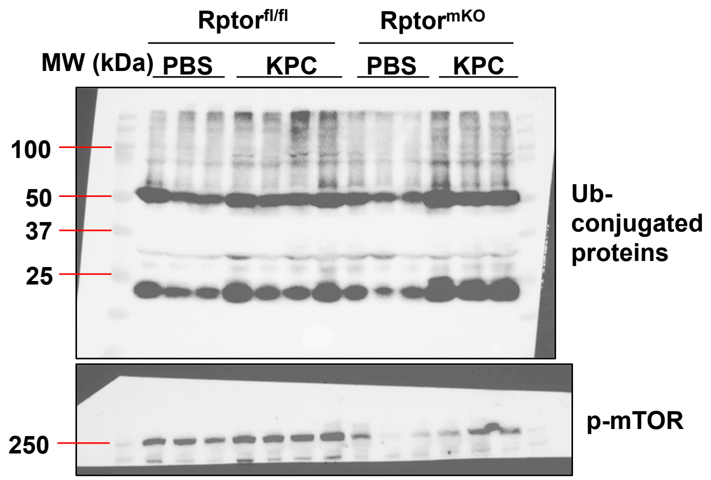

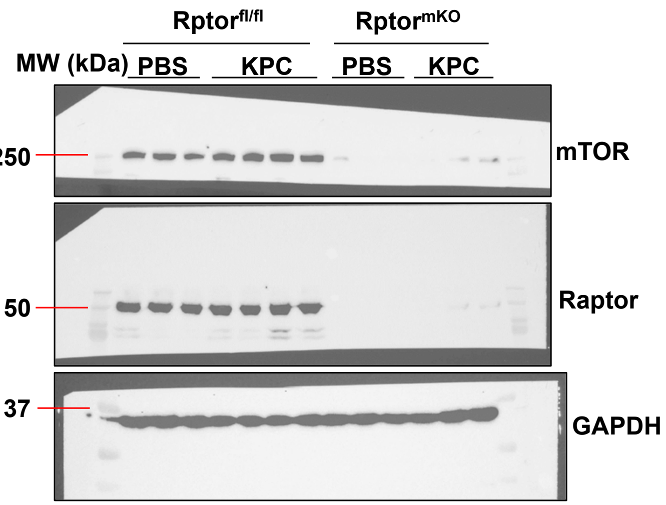

Supplement: Unedited blot and gel images [file jciinsight-11-200396-s347.pdf]
